# Proteomic analyses do not reveal subclinical inflammation in fatigued patients with clinically quiescent inflammatory bowel disease

**DOI:** 10.1038/s41598-022-17504-5

**Published:** 2022-08-26

**Authors:** Arno R. Bourgonje, Sietse J. Wichers, Shixian Hu, Hendrik M. van Dullemen, Marijn C. Visschedijk, Klaas Nico Faber, Eleonora A. M. Festen, Gerard Dijkstra, Janneke N. Samsom, Rinse K. Weersma, Lieke M. Spekhorst

**Affiliations:** 1 Department of Gastroenterology and Hepatology, University of Groningen, University Medical Center Groningen, P.O. Box 30.001, 9700 RB Groningen, The Netherlands; 2 Department of Genetics, University of Groningen, University Medical Center Groningen, Groningen, The Netherlands; 3 Division of Gastroenterology, Department of Pediatrics, Erasmus University Medical Center, Rotterdam, The Netherlands; 4 Department of Gastroenterology and Hepatology, Medisch Spectrum Twente, Enschede, The Netherlands

**Keywords:** Gastroenterology, Inflammatory bowel disease, Proteomics, Fatigue

## Abstract

Fatigue is a common and clinically challenging symptom in patients with inflammatory bowel diseases (IBD), occurring in ~ 50% of patients with quiescent disease. In this study, we aimed to investigate whether fatigue in patients with clinically quiescent IBD is reflected by circulating inflammatory proteins, which might reflect ongoing subclinical inflammation. Ninety-two (92) different inflammation-related proteins were measured in plasma of 350 patients with clinically quiescent IBD. Quiescent IBD was defined as clinical (Harvey-Bradshaw Index < 5 or Simple Clinical Colitis Activity Index < 2.5) and biochemical remission (C-reactive protein < 5 mg/L and absence of anemia) at time of fatigue assessment. Leukemia inhibitory factor receptor (LIF-R) concentrations were inversely associated with severe fatigue, also after adjustment for confounding factors (nominal *P* < 0.05). Although solely LIF-R showed weak ability to discriminate between mild and severe fatigue (area under the curve [AUC] = 0.61, 95%CI: 0.53–0.69, *P* < 0.05), a combined set of the top seven (7) fatigue-associated proteins (all *P* < 0.10) was observed to have reasonable discriminative performance (AUC = 0.82 [95%CI: 0.74–0.91], *P* < 0.01). Fatigue in patients with IBD is not clearly reflected by distinct protein signatures, suggesting there is no subclinical inflammation defined by the studied inflammatory proteins. Future studies are warranted to investigate other proteomic markers that may reflect fatigue in clinically quiescent IBD.

## Introduction

Inflammatory bowel diseases (IBD), encompassing Crohn’s disease (CD) and ulcerative colitis (UC), are chronic relapsing diseases of the gastrointestinal (GI) tract. Although the exact origin is not fully understood, a complex interplay between genetics, environmental factors, the gut microbiota, and host immune system is believed to underlie disease pathogenesis^1^. Clinically, IBD is characterized by alternating periods of active and quiescent disease, which remain difficult to predict and a challenge to treat. Major clinical symptoms include abdominal pain, abnormal stool frequency and consistency, weight loss, and fatigue. In many patients, the latter symptom is notoriously persistent, and affects both patients with active disease and those who are in clinical remission^2^.

Fatigue is a frequently occurring, multi-dimensional, but understudied phenomenon in patients with IBD, posing a great challenge for treating physicians^3^. Persistent fatigue is negatively associated with quality of life (QoL), is accompanied by lower stress tolerance, lower resilience to overexhaustion, and it is associated with lower rates of socio-economic participation^4,5^. The prevalence of fatigue in IBD is high, occurring in up to 80% of patients with active disease and in approximately 50% of patients having quiescent disease^6^.

Previous studies have linked inflammatory disease activity to fatigue in various chronic diseases, including IBD, but also in cancer, rheumatic- and autoimmune diseases^3,7^. In patients with IBD, the severity of fatigue has been associated with higher disease activity as measured by clinical disease activity scores^8,9^. In contrast, other studies have demonstrated that fatigue is not dependent on disease activity, and that it may even increase in patients who are in remission^10,11^. Despite the fact that the association between fatigue and disease activity remains poorly defined, it is evident that many patients with IBD who are in remission still suffer from fatigue that often persists over time. However, the multifactorial etiology of fatigue in IBD makes it difficult to pinpoint the exact origin(s), impeding the initiation of appropriate and effective treatment^12^. Potential contributing factors may include, among others, nutritional deficiencies, medical and psychological comorbidity, sleep disturbances, or a subclinical pro-inflammatory state^13^.

Considering the potential association between subclinical inflammation and chronic fatigue in patients with IBD, fatigue may be characterized by distinct (inflammatory) protein signatures^14,15^. This may lead to the identification of key molecular players in the pathophysiology of fatigue, which may, in turn, expose potential therapeutic targets in order to improve management of fatigue in patients with IBD. For example, a previous study found that systemic concentrations of pro-inflammatory cytokines IFN-γ, TNF-α, IL-12 as well as numbers of memory T-cells and neutrophils were higher among fatigued patients with clinically quiescent IBD, whereas IL-6 and monocyte concentrations were lower^14^. In contrast, a recent prospective multi-omics-based study that included proteomics data did not detect marked differences in inflammatory proteins when comparing fatigued and non-fatigued patients who had clinically and endoscopically quiescent IBD^15^. However, only few of such studies have been performed to characterize the potential subclinical pro-inflammatory state in fatigued patients with clinically quiescent IBD, and current findings are often contrasting each other.

In this study, we aimed to study associations between plasma inflammatory proteins and fatigue severity in patients with clinically and biochemically quiescent IBD. To do so, we leveraged proteomics technology targeted at inflammation-related plasma proteins in order to characterize the potential presence of a subclinical pro-inflammatory state.

## Results

### Cohort characteristics

In total, 350 patients were included, of which 188 patients were diagnosed with CD and 162 patients with UC. Patients with the lowest fatigue scores (belonging to the lowest 25% or 1st quartile, Q1) and highest fatigue scores (belonging to the top 25% or 4th quartile, Q4) were compared for their demographic and clinical characteristics (Table 1). The proportion of patients with CD among those reporting the highest fatigue scores (Q4) was higher compared to patients with low fatigue scores (Q1) (63.6% vs. 50.0%, *P* = 0.06), while the proportion of patients with UC was correspondingly lower (36.4% vs. 50.0%). The proportion of females was higher among fatigued patients (*P* < 0.01). Age and body-mass index (BMI) distributions were similar between lowest (Q1) and highest (Q4) fatigued patients (*P* = 0.95 and *P* = 0.16, respectively). Patients with highest fatigue scores smoked more often compared to patients with low fatigue scores (*P* < 0.01). No differences were observed between patients having the lowest (Q1) vs. highest (Q4) fatigue scores with regard to the use of thiopurines (*P* = 0.90), TNF-α-antagonists (*P* = 0.25) or other types of IBD medications (Table 1). A comparison of cohort characteristics of the full study population, comparing patients with below-median and above-median (Q1-2 vs. Q3-4) fatigue scores can be found in Supplementary Table S1.

**Table 1:** Demographic and clinical characteristics of the study population compared between mildly fatigued (Q1) and severely (Q4) fatigued patients.

Variable	Total	Q1 fatigue score (0–3)	Q4 fatigue score (6–10)	*P*-value
	*n* = 195	*n* = 96	*n* = 99	
Age (years)	40.5 ± 14.4	40.5 ± 14.8	40.6 ± 14.2	0.95
Sex, *n* (%)				< 0.01
Male	104 (53.3)	64 (66.7)	40 (40.4)	
Female	91 (46.7)	32 (33.3)	59 (59.6)	
BMI (kg/m^2^)	24.4 [22.2;26.9]	24.0 [22.3;26.3]	24.7 [22.2;27.9]	0.16
IBD diagnosis, *n* (%)				0.06
CD	111 (56.9)	48 (50.0)	63 (63.6)	
UC	84 (43.1)	48 (50.0)	36 (36.4)	
Current smoking, *n* (%)	186 (95.4)	89 (92.7)	97 (98.0)	< 0.01
Yes	41 (21.0)	11 (12.4)	30 (30.9)	
No	145 (74.4)	78 (87.6)	67 (69.1)	
**Montreal classification**				
Montreal Age (A), *n* (%)	194 (99.5)	96 (100)	98 (99.0)	0.47
A1 (≤ 16 years)	31 (15.9)	18 (18.8)	13 (13.3)	
A2 (17–40 years)	129 (66.2	60 (62.5)	69 (70.4)	
A3 (> 40 years)	34 (17.4)	18 (18.8)	16 (16.3)	
Montreal Location (L), *n* (%)	111 (100)	48 (100)	63 (100)	0.24
L1 (ileal disease)	39 (35.1)	16 (33.3)	23 (36.5)	
L2 (colonic disease)	25 (22.5)	14 (29.2)	11 (17.5)	
L3 (ileocolonic disease)	38 (34.2)	13 (27.1)	25 (39.7)	
L4 (upper GI disease)	2 (1.8)	1 (2.1)	1 (1.6)	
L1 + L4	4 (3.6)	1 (2.1)	3 (4.8)	
L2 + L4	2 (1.8)	2 (4.2)	0 (0.0)	
L3 + L4	1 (0.9)	1 (2.1)	0 (0.0)	
Montreal Behavior (B), *n* (%)	111 (100)	48 (100)	63 (100)	0.21
B1 (non-stricturing, non-penetrating)	54 (48.6)	19 (39.6)	35 (55.6)	
B2 (stricturing)	20 (18.0)	9 (18.8)	11 (17.5)	
B3 (penetrating)	10 (9.0)	7 (14.6)	3 (4.8)	
B1 + P (perianal disease)	5 (4.5)	3 (6.3)	2 (3.2)	
B2 + P (perianal disease)	14 (12.6)	8 (16.7)	6 (9.5)	
B3 + P (perianal disease)	8 (7.2)	2 (4.2)	6 (9.5)	
Montreal Extension (E), *n* (%)	84 (100)	48 (100)	36 (100)	0.44
E1 (proctitis)	16 (19.0)	7 (14.6)	9 (25.0)	
E2 (left-sided colitis)	23 (27.4)	13 (27.1)	10 (27.8)	
E3 (pancolitis)	45 (53.6)	28 (58.3)	17 (47.2)	
**Medication use**				
Aminosalicylates, *n* (%)	65 (33.3)	34 (35.4)	31 (31.3)	0.54
Thiopurines, *n* (%)	76 (39.0)	37 (38.5)	39 (39.4)	0.90
Steroids, *n* (%)	28 (14.4)	18 (18.8)	10 (10.1)	0.09
Calcineurin inhibitors, *n* (%)	6 (3.1)	5 (5.2)	1 (1.0)	0.12
Methotrexate, *n* (%)	6 (3.1)	2 (2.1)	4 (4.0)	0.68
TNF-α-antagonists, *n* (%)^†^	33 (16.9)	13 (13.5)	20 (20.2)	0.25
**Surgical history**				
Ileocecal resection, *n* (%)	35 (82.1)	17 (17.7)	18 (18.2)	0.93
Colon resection (or partial), *n* (%)	26 (13.3)	10 (10.4)	16 (16.2)	0.24
**Laboratory parameters**				
Hb (mmol/L) (males)	9.4 [9.0–9.8]	9.4 [9.1–9.7]	9.6 [9.0–9.9]	0.47
Hb (mmol/L) (females)	8.3 [7.9–8.8]	8.5 [7.8–8.9]	8.3 [7.9–8.7]	0.37
CRP (mg/L)*	5.0 [1.4–5.0]	3.9 [0.9–5.0]	5.0 [1.9–5.0]	0.05

Data are presented as proportions *n* with corresponding percentages (%) or as median [interquartile range, IQR] in case of continuous variables. *P*-values ≤ 0.05 were considered statistically significant.

*BMI* body-mass index, *CD* Crohn’s disease, *IBD* inflammatory bowel disease, *TNF-α* tumor necrosis factor alpha, *UC* ulcerative colitis.

*Numericized lower limits of detection (< 5 mg/L) were included in calculating median and IQR, but may falsely represent the (unknown) true biological value. All CRP values were < 5 mg/L, as this was one of the study’s inclusion criteria.

^†^The use of TNF-α-antagonists included use of the following compounds: infliximab, adalimumab, golimumab and certolizumab pegol.

### Distributions of fatigue and psychological well-being scores

**Figure 1:**
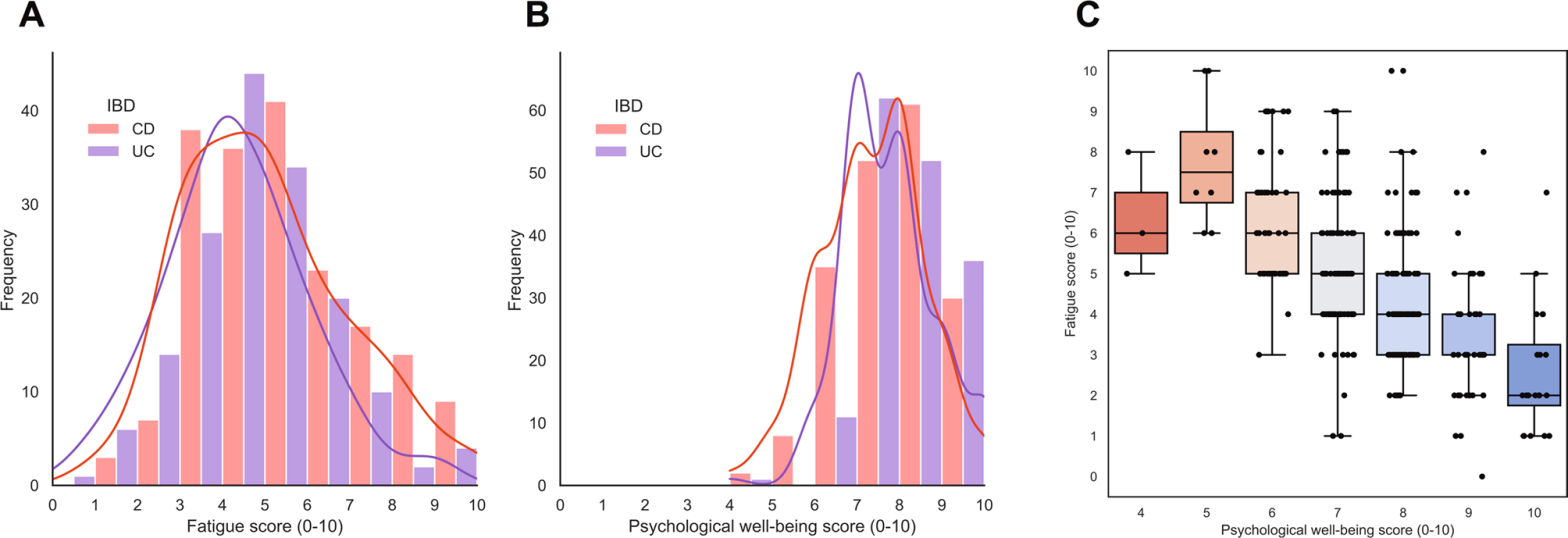
(**A**) Patient-reported fatigue scores follow a rather normal distribution for patients with CD (red) and UC (purple). (**B**) Patient-reported psychological well-being scores show a negative (left-) skewed distribution. (**C**) Boxplots of fatigue scores plotted against psychological well-being scores, demonstrating that fatigue inversely correlates with psychological well-being.

Median fatigue scores among patients with CD and UC were 5 [IQR: 3–6] and 4 [IQR: 3–5], and median psychological well-being scores were 7 [IQR: 7–8] and 8 [IQR: 7–8], respectively (Fig. 1). Fatigue was normally distributed across the full study cohort, as well as for CD and UC separately, whereas scores for psychological well-being were negatively (left-)skewed (Fig. 1A, B). Fatigue scores were significantly inversely associated with psychological well-being scores (Spearman’s rho (*⍴*): − 0.58, *P* < 0.001) (Fig. 1C).

### Associations of plasma protein concentrations and fatigue scores in patients with IBD

**Figure 2:**
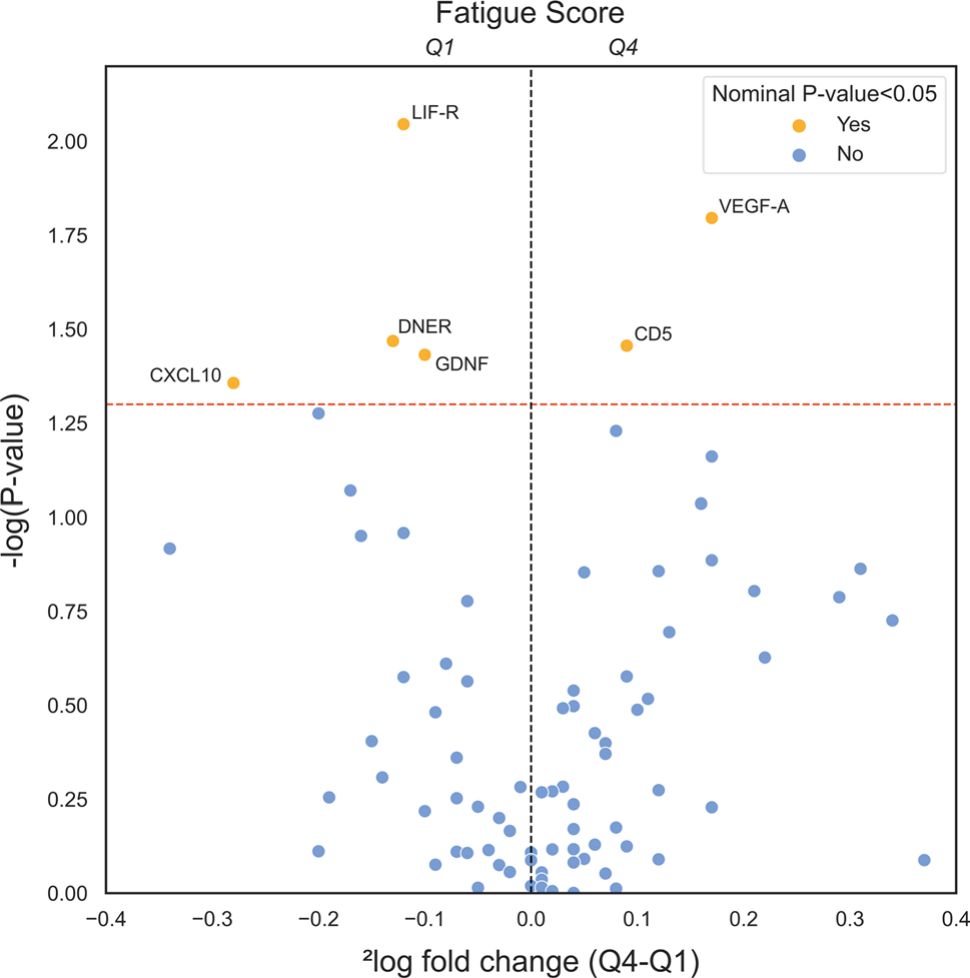
Volcano plot demonstrating differentially abundant plasma proteins between patients with the lowest (Q1, range 0–3) and highest (Q4, range 6–10) fatigue scores. The red horizontal dashed line indicates the threshold for nominal significance (nominal *P* < 0.05), and the vertical black dashed line indicates zero difference between the groups. Abbreviations: Q1, first quartile; Q4, fourth quartile.

**Figure 3:**
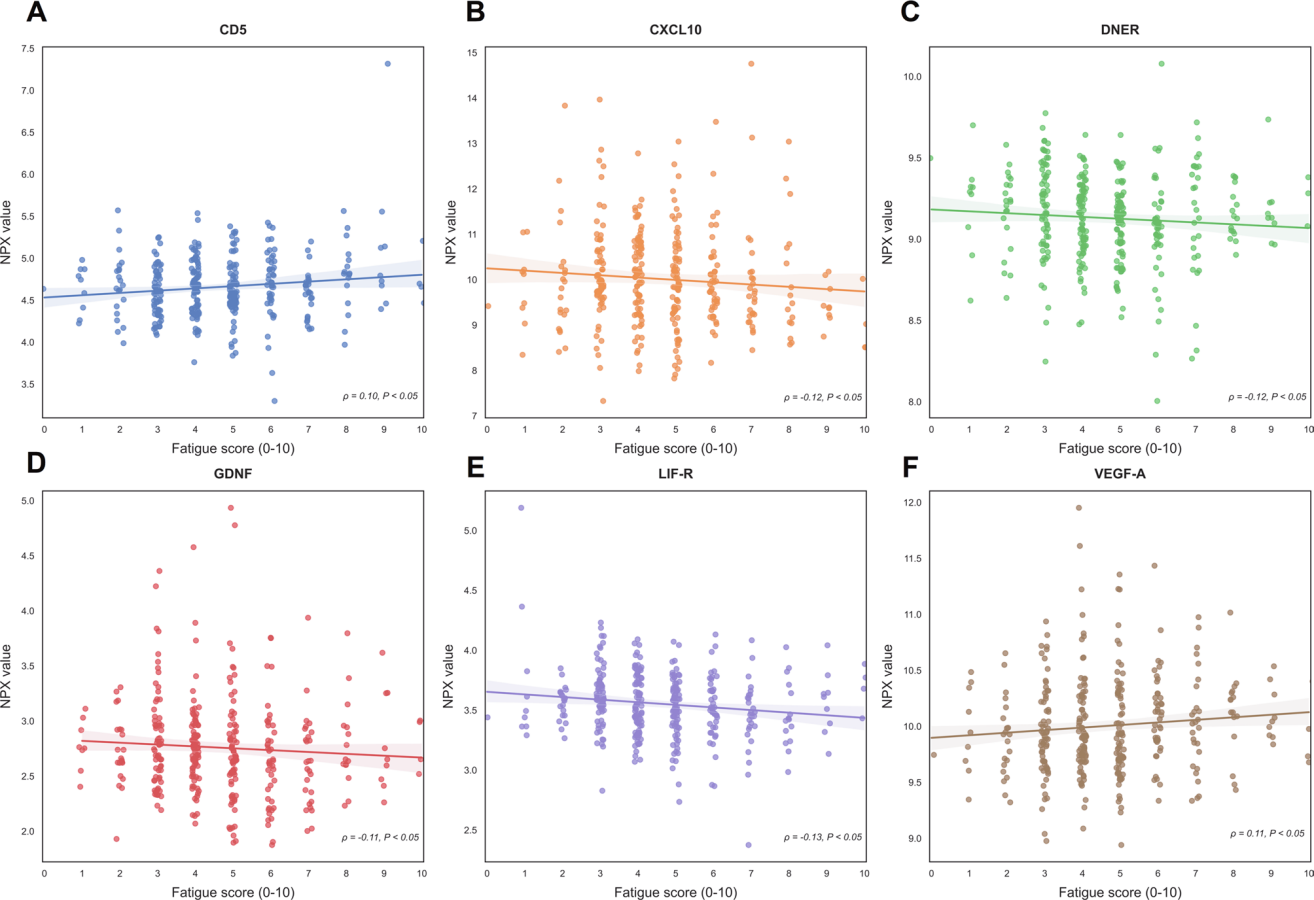
(**A**–**F**) Top six (6) differentially abundant plasma proteins between mildly fatigued (Q1) patients and severely fatigued (Q4) patients. Abbreviations: CD5, T-cell surface glycoprotein CD5; CXCL10, C-X-C motif chemokine ligand 10; DNER, Delta and Notch-like epidermal growth factor-related receptor; GDNF, glial cell line-derived neurotrophic factor; leukemia inhibitory factor receptor; NPX, normalized protein expression; vascular endothelial growth factor A (VEGF-A).

Plasma protein concentrations were compared between patients with lowest fatigue scores (Q1, ranging from 0 to 3, *n* = 96) and patients with the highest fatigue scores (Q4, ranging from 6 to 10, *n* = 99) (Supplementary Table S2). None of the quantified plasma proteins concentrations were found to be differentially abundant after adjusting for multiple comparisons (Benjamini–Hochberg method, all FDR > 0.1). However, six (6) plasma proteins were observed to be nominally significant (*P* < 0.05). Among these proteins, leukemia inhibitory factor receptor (LIF-R), Delta and Notch-like epidermal growth factor-related receptor (DNER), glial cell line-derived neurotrophic factor (GDNF), and C-X-C motif chemokine ligand 10 (CXCL10) were lower in fatigued (Q4) patients, whereas concentrations of vascular endothelial growth factor A (VEGF-A) and T-cell surface glycoprotein CD5 (CD5) were found to be relatively higher in fatigued (Q4) patients (Figs. 2 and 3). The same analyses were repeated in patients with below-median (ranging from 0 to 4, *n* = 176) compared to above-median (ranging from 5 to 10, *n* = 174) fatigue scores (Supplementary Table S3), which showed fairly similar results.

After subdividing the cohort into patients with CD and UC and creating CD- and UC-specific quartiles of fatigue scores, plasma proteins were compared again between patients with Q1 (range: 0–3 for both CD and UC) and Q4 (CD: 7–10; UC: 6–10) fatigue scores (Supplementary Tables S4 and S5). Similar to the full cohort analysis, no plasma proteins were observed to be differentially abundant after adjustment for multiple comparisons (FDR > 0.1). Among patients with CD, plasma concentrations of extracellular newly identified receptor for advanced glycation endproducts binding protein (EN-RAGE, also known as S100A12 or calgranulin C) and GDNF were differentially abundant under nominal significance (*P* < 0.05), while among patients with UC, neurturin (NRTN), adenosine deaminase (ADA) and fibroblast growth factor (FGF-23) were nominally significant (*P* < 0.05) (Supplementary Fig. S1).

Amongst the nominally significant proteins in the full cohort analysis, only GDNF was associated with the psychological well-being score (Spearman’s *⍴* = 0.16, *P* < 0.05), while the remaining proteins showed weak, non-significant correlations (Supplementary Fig. S2).

### Leukemia inhibitory factor receptor (LIF-R) is most strongly associated with fatigue

Subsequently, logistic regression analyses were performed to evaluate associations between plasma proteins and the presence of fatigue in patients with IBD, while having the possibility to control for potential confounding factors. In univariable logistic regression analysis, proteins were identified that were associated with the presence of fatigue while adopting a pre-selection threshold of *P* < 0.10 (Table 2). Multivariable analysis revealed gender and smoking behavior as relevant confounding factors, and when adjusted for these factors, an inverse association between plasma LIF-R concentrations and the presence of severe fatigue (*P* < 0.05) was observed (Q4 vs. Q1). All remaining proteins from univariable analysis were not significantly associated with fatigue after adjustment for confounding factors.

**Table 2:** Univariable and multivariable logistic regression analyses for associations between plasma proteins and the presence or absence of fatigue in patients with IBD (defined as by median or by the lowest (Q1) versus highest quartile (Q4) of fatigue scores).

Protein	Univariable analysis		Multivariable analysis	
	OR (95% CI)	*P*-value	aOR (95% CI)*	*P*-value
**Q1 versus Q4 fatigue scores**				
LIF-R	0.21 (0.07–0.63)	0.005	0.29 (0.09–0.94)	**0.039**
VEGF-A	2.42 (1.15–5.07)	0.020	2.14 (0.97–4.73)	0.061
GDNF	0.49 (0.24–0.97)	0.041	0.72 (0.34–1.51)	0.388
IL-20RA	4.72 (1.02–21.9)	0.048	4.24 (0.76–23.7)	0.100
DNER	0.39 (0.15–1.02)	0.055	1.12 (0.38–3.33)	0.839
CD5	2.16 (0.99–4.49)	0.052	2.32 (0.99–5.45)	0.053
EN-RAGE	0.64 (0.39–1.06)	0.084	0.78 (0.46–1.35)	0.376

Data are presented as odds ratios (ORs) with corresponding 95% confidence intervals (CI) and *P*-values. Bold *P*-values indicate nominal *P*-values < 0.05 in multivariable analysis.

*OR* odds ratio, *CI* confidence interval, *aOR* adjusted odds ratio.

*Significant confounding variables included in multivariable analysis were gender (male/female) and current smoking (no/yes).

To evaluate the discriminative capacity of plasma proteins that were incorporated into logistic regression analyses, receiver operating characteristics (ROC) statistics were calculated with the corresponding area under the curve (AUC) as overall measure of fit (Table 3). Individual plasma proteins demonstrated almost no discriminative performance with regard to the presence of severe (Q4) fatigue compared with mild (Q1) fatigue. Among these proteins, the LIF-R protein demonstrated the highest, but still very weak discriminative value (AUC 0.61 [95% CI: 0.53–0.69] for Q1 vs. Q4, *P* < 0.01, Supplementary Fig. S3), which slightly dropped after cross-validation (cv-AUC: 0.59 [95% CI: 0.53–0.65]).

**Table 3:** Receiver operating characteristics (ROC) analysis demonstrating the discriminative value of plasma proteins with regard to the presence of (severe) fatigue in patients with IBD, defined by the lowest (Q1) versus highest (Q4) quartile of fatigue scores.

Protein	AUC (95% CI)	CV-AUC (95% CI)	*P*-value
**Q1 versus Q4 fatigue scores**			
LIF-R	0.61 (0.53–0.69)	0.59 (0.53–0.65)	< 0.01
VEGF-A	0.60 (0.52–0.68)	0.61 (0.54–0.68)	0.016
GDNF	0.59 (0.51–0.67)	0.60 (0.50–0.70)	0.037
IL-20RA	0.61 (0.50–0.73)	0.59 (0.45–0.73)	0.059
DNER	0.59 (0.51–0.67)	0.57 (0.50–0.64)	0.034
CD5	0.59 (0.51–0.67)	0.58 (0.51–0.65)	0.035
EN-RAGE	0.58 (0.50–0.66)	0.58 (0.51–0.65)	0.053
Combination	0.82 (0.74–0.91)	0.79 (0.73–0.85)	< 0.01

Data are presented as areas under the curve (AUC) and AUCs after cross-validation (CV-AUC) with corresponding 95% confidence intervals (CI) and *P*-values.

*AUC* area under the curve, *CV-AUC* cross-validated area under the curve, *CI* confidence interval.

### Combinations of plasma proteins demonstrate reasonable discriminative performance

**Figure 4:**
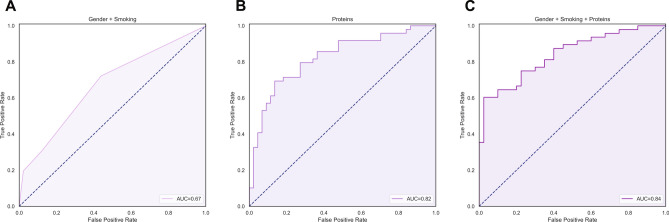
(**A**–**C**) Receiver operating characteristics (ROC) curves showing the discriminative value of (**A**) gender and smoking as relevant confounding factors and (**B**) a combined panel of seven (7) proteins (consisting of CD5, DNER, EN-RAGE, GDNF, IL-20RA, LIF-R, and VEGF-A) with regard to the presence of fatigue in patients with IBD. (**C**) When combining the seven proteins with gender and smoking as confounding factors, the classification performance increased. Abbreviations: AUC, area under the curve; Q1, first quartile; Q4, fourth quartile.

Next, we aimed to evaluate the combined predictive value of all seven (7) plasma proteins that were incorporated into logistic regression analyses (*P* < 0.10) and subsequent ROC analyses (Tables 2 and 3) with regard to the presence of fatigue (Fig. 4). First, the discriminative capacity of the identified confounding factors (i.e., gender and smoking) was established, which demonstrated weak performance (AUC = 0.67, 95% CI: 0.60–0.75, *P* < 0.01). Second, when combining the seven proteins (LIF-R, VEGF-A, GDNF, IL-20RA, DNER, CD5 and EN-RAGE) that were analysed in relation to the lowest (Q1) vs. highest (Q4) fatigue quartiles, reasonable discriminative performance could be achieved (AUC = 0.82 [95% CI: 0.74–0.91], *P* < 0.01, Fig. 4B), which did not materially change after cross-validation (AUC = 0.79 [95% CI: 0.73–0.85]). Finally, when this panel of seven proteins was combined with confounding factors, the discriminative performance further improved (cross-validated AUC = 0.84, 95% CI: [0.76–0.92], *P* < 0.01, Fig. 4C).

## Discussion

This study demonstrates that inflammatory proteins do not strongly associate with fatigue in patients with clinically quiescent IBD. Although we failed to detect differentially abundant plasma proteins between fatigued and non-fatigued patients, some potentially relevant proteomic signals were observed, albeit characterized by weak associations and only nominal statistical significance. Leukemia inhibitory factor receptor (LIF-R) was the top associated plasma protein in relation to fatigue scores, also after adjustment for gender and smoking as relevant confounders. Individual plasma proteins showed weak discriminative capacity between mildly (Q1) and severely (Q4) fatigued patients, whereas a combined set of seven top associated proteins showed reasonable discriminative performance. Collectively, these findings imply that fatigue is not solely driven by overt subclinical inflammation and that individual inflammatory proteins do not serve as accurate biomarkers, which could aid in quantification of fatigue burden or expose novel avenues for therapeutic intervention.

Fatigue is one of the most common and disabling symptoms of patients with IBD, and occurs in approximately half of patients with quiescent disease^6^. Since its multifactorial etiology remains elusive and its assessment rather subjective, there is an urgent need for fatigue biomarkers. Multiple studies have demonstrated that fatigue is associated with clinically active IBD and systemic inflammation^11,16,17^. In contrast, there are few examples of studies that focused on the potential role of a subclinical pro-inflammatory state in fatigued patients with clinically quiescent IBD. In a study that analysed immune parameters in relation to fatigue in patients with clinically quiescent IBD, systemic concentrations of pro-inflammatory cytokines IFN-γ, TNF-α, IL-12 as well as numbers of memory T-cells and neutrophils were higher among fatigued patients, whereas IL-6 and monocyte concentrations were lower^14^. Similarly, raised pro-inflammatory cytokine concentrations (IFN-γ, IL-6, IL-12, IL-17A) were reported in relation to fatigue in a pediatric IBD population^18^. In contrast, another study observed no significant differences in inflammatory markers (including CRP, calprotectin, IFN-γ, IL-6, IL-1β, TNF-α, IL-12, IL-17A) between fatigued and non-fatigued patients who were in deep remission, which is more in line with our findings^17^. Likewise, no associations were observed between CRP, IL-5, IL-8 and IL-12 concentrations and fatigue scores among 202 patients with IBD who were in clinical remission^19^. Furthermore, a recent prospective multi-omics-based study that included proteomics data did not detect marked differences in inflammatory proteins when comparing fatigued and non-fatigued patients with clinically and endoscopically quiescent IBD^15^. Despite the clear observations that active IBD, as reflected by elevated concentrations of inflammatory markers, is significantly correlated with fatigue severity, fatigue in IBD is not necessarily solely driven by subclinical inflammation.

In the present study, LIF-R was the top associated plasma protein in relation to fatigue scores. LIF-R (CD118) is a receptor for leukemia inhibitory factor (LIF) and IL-6-like cytokines, and interacts with a high-affinity subunit, glycoprotein 130 (gp130), which act together in the oncostatin M (OSM) signaling pathway. LIF is a pleiotropic cytokine that affects proliferation, maturation and survival of a wide variety of body cells^20^. Heterodimerization of LIF-R or the OSM-specific receptor (OSM-R) with gp130 activates OSM, which is considered as an ‘inflammatory amplifier’ and drives intestinal inflammation in IBD (mainly by activation of JAK-STAT and PI3K-Akt pathways), leading to increased production of cytokines, chemokines and adhesion molecules^20,21^. OSM is also a marker for non-responsiveness to TNF-α-antagonists in patients with IBD^22^. Mucosal LIF-R expression has been shown to be decreased in biopsies of newly diagnosed IBD patients as well as in patients without endoscopic remission^21^. Therefore, it could be hypothesized that low circulating LIF-R concentrations, which may reflect subclinical inflammation, drive fatigue through modification of the pro-inflammatory response. This, however, should theoretically be accompanied by higher concentrations of LIF, which we were not able to investigate in the present study due to a very low detection rate (< 10%). LIF and LIF-R have also been associated with fatigue beyond IBD. For instance, LIF is known for its strong cachexia-inducing ability in animal models, whereas it is also associated with cancer-related fatigue and weight loss in humans^23,24^. In addition, decreased activity of LIF-R but increased LIF activity have been associated with neuro-inflammation^25^. LIF-R plays a crucial role in enhancing cellular survival of neural cells and confers anti-inflammatory effects via stimulation of other neuroprotective cytokines. Considering this, reduced shedding of the LIF-R protein might be related to fatigue in IBD through induction of pro-inflammatory phenotypes of T-lymphocytes, macrophages, or microglia.

In attempting to characterize an inflammatory protein signature for fatigue in patients with clinically quiescent IBD, we found a combination of LIF-R, VEGF-A, GDNF, IL-20RA, DNER, CD5 and EN-RAGE to have reasonable ability in discriminating between mild and severe fatigue.

Fatigued patients exhibited lower concentrations of glial-derived neurotrophic factor (GDNF) and Delta and Notch-like epidermal growth factor-related receptor (DNER). The observation of lower GDNF concentrations in fatigued patients may potentially reflect the disturbed intestinal barrier function as is characteristic for patients with IBD. In a murine colitis model, GDNF was shown to ameliorate intestinal epithelial barrier function through reducing epithelial permeability and inhibiting mucosal inflammation^26^. Similarly, GDNF concentrations were previously found to be decreased in the inflamed intestine of patients with IBD, where GDNF attenuated desmoglein 2 (DSG2)-associated impairment of intestinal barrier function^27^. Similar to GDNF, plasma DNER concentrations were decreased in severely fatigued patients compared with mildly fatigued patients. Previously, we demonstrated that DNER concentrations are strongly inversely associated with CRP concentrations^28^. DNER activates the Notch-1 signaling pathway, which is associated with improved mucosal barrier function^29^. As opposed to GDNF and DNER, plasma VEGF-A concentrations were increased in fatigued patients. VEGF-A is a well-known mediator in IBD by stimulating intestinal inflammation, angiogenesis and leukocyte adhesion^30^. Active IBD is characterized by increased VEGF-A concentrations in the blood and the inflamed intestinal mucosa^30^. Importantly, intestinal neovascularization in IBD occurs in a notoriously disorganized manner, and the newly formed blood vessels are highly permeable as is evident by associated edema^31^. This may lead to disruption of the intestinal endothelial barrier, which may also result in compromised epithelial barrier integrity via angiocrine communication^32^. Given the above considerations, one may speculate that these protein alterations in fatigued patients reflect impaired intestinal barrier function, which could in turn reflect subclinical inflammation and give rise to fatigue perception through gut-brain axis signaling.

Strengths of the present study include the extensive phenotypic characterization of the study cohort, together with an exact time matching (within 24 h) of sampling and fatigue assessment. Furthermore, we were able to select a large patient cohort only consisting of patients with clinical (HBI or SCCAI scores) and biochemical (CRP) remission in the absence of anemia. There are also several limitations to this study that warrant recognition. For example, no endoscopic assessments of disease activity or fecal calprotectin levels were available at time of sampling, which would have preferentially been used to confirm the quiescent or active state of the disease. Instead, we had to rely on clinical and serological assessment of disease activity, which necessitates cautious interpretation as some included patients might have had subclinical intestinal inflammation. Second, this study was of cross-sectional design and did not include follow-up data, which could have enabled us to investigate fatigue and protein concentration trajectories and thereby unravel the dynamics of the observed associations. Third, fatigue was assessed over a period of 24 h, whereas this is not an accurate representation of chronic fatigue that is experienced by patients with IBD having either quiescent or active disease. Likewise, psychological well-being was also assessed over a period of 24 h, which does not take into account other potentially existing co-morbidities such as mood disturbances, chronic anxiety, or the experience of stressful life-events. In addition, both scores were only assessed in a cohort of patients with IBD, but their distributions were not assessed in unaffected population controls. Furthermore, some information was missing that could potentially affect fatigue in these patients, including medical and psychological comorbidity relevant to fatigue, nutritional deficiencies, sleep quality, and more granular information on psychological well-being. Finally, no absolute protein quantification was performed as this would have been possible by using more traditional methods such as enzyme-linked immunosorbent assays (ELISAs). This limits the possibility to compare protein concentrations between each other or with concentrations reported in previous studies. Instead, relative protein quantification was achieved through PEA technology, which is accompanied by high sensitivity and high precision compared to other multiplex proteomics techniques.

Our results indicate that inflammation-related plasma proteins may not be the best proteomic or metabolic markers for defining biomarker signatures for fatigue in patients with clinically quiescent IBD. Instead, markers representing alternative pathophysiological mechanisms or biological systems may be more promising to further investigate. For example, a recently published explorative study found alterations in plasma lipid profiles reflecting fatigue in IBD, which were mainly characterized by disturbances within the arachidonic acid and sphingolipid pathways^33^. Another study has linked alterations in the gut microbiome and serum metabolome to persistent fatigue in patients with quiescent IBD, which supported the prevailing gut-brain axis hypothesis in fatigue pathophysiology^15^.

This study aimed to contribute to the need for defining biomarkers for fatigue, being an important and clinically challenging symptom in patients with IBD. Until date, only few studies focused on the pathophysiological mechanisms that may underlie fatigue in the context of IBD, whereas these efforts in elucidating mechanisms were rather limited to specific and well-characterized disease entities such as chronic fatigue syndrome (CFS) and fibromyalgia^15^. To that end, our results may also have implications for fatigue beyond IBD, as fatigue is also prevalent among various other autoimmune diseases, such as rheumatoid arthritis or multiple sclerosis^34,35^. Therefore, a broader search for biomarker signatures for fatigue merits further research in a variety of clinical contexts and even beyond pre-defined disease entities.

In conclusion, this study provides evidence that systemic inflammation, as reflected by circulating inflammatory proteins, may not be the primary driver of fatigue in patients with clinically quiescent IBD. Further, the LIF-R protein may potentially be involved in the pathophysiology of fatigue in IBD, which requires further validation. Future studies are warranted to investigate the potential of other proteins to quantify fatigue burden in patients with clinically quiescent IBD, preferably proteins that represent alternative pathophysiological pathways.

## Methods

### Study population

Patients with an established diagnosis of IBD were included at the outpatient clinic of the University Medical Center Groningen (UMCG), Groningen, the Netherlands. Patients were included based on their participation in the 1000IBD project: a large, deeply phenotyped cohort consisting of over 1,000 patients with IBD living in the northern parts of the Netherlands^36^. Patients were enrolled in the 1000IBD project in the period from 2010 to 2019. In this study, patients were included when they were classified as being in clinical and biochemical remission at the time of their visit. Patients having anemia were excluded from the study. The study was approved by the Institutional Review Board (IRB) of the UMCG (registered as no. 08/338). All patients provided written informed consent for their participation in the study. The study has been performed in accordance with the principles of the Declaration of Helsinki (2013).

### Data collection

Detailed information on demographic and clinical variables was registered for all patients, including age, sex, body-mass index (BMI), smoking behavior, Montreal disease classification, medication usage, history of bowel surgery, and disease activity. All this information was assessed at the same time when plasma samples were collected for proteomic profiling. Clinical disease activity was assessed using the Harvey-Bradshaw Index (HBI) for patients with CD and the Simple Clinical Colitis Activity Index for patients with UC. Blood hemoglobin and C-reactive protein (CRP) concentrations were routinely measured as part of clinical care on the exact same date of plasma sampling.

### Study outcomes and definitions

The primary study outcome was the subjective assessment of fatigue, which is part of the IBD-specific outpatient assessment in the UMCG and was evaluated by a single item during patients’ visits to the outpatient clinic at the same date of plasma sampling: “*How fatigued has the patient been during the last 24 h?*” (in Dutch: “*In hoeverre heeft u last gehad van vermoeidheid in de afgelopen 24 uur?*”)^8^ Answers to this item were based upon a visual analogue 10-point Likert scale, ranging from a score of 1 (being not fatigued at all) to 10 (being severely fatigued) (Supplementary Fig. S4). The secondary study outcome was an assessment of psychological well-being within 24 h, which was evaluated by the following item: “*How does the patient feel [during the last 24 h]*?” (in Dutch: “*Welk rapportcijfer geeft u uw algemeen welzijn over de afgelopen 24 uur?*”), and rated in a similar manner based on a visual analogue 10-point Likert scale, ranging from 1 (very bad) to 10 (excellent). Clinical remission was defined as an HBI < 5 for CD or SCCAI < 2.5 for UC^37^. Biochemical remission was defined as serum C-reactive protein (CRP) concentration < 5 mg/L. Anemia was defined as a hemoglobin concentration < 8.5 mmol/L for men and < 7.5 mmol/L for women, based on the Dutch national reference ranges^38^.

### Proteomic profiling

Plasma concentrations of 92 different inflammation-related proteins were quantified using proximity extension assay (PEA) technology using the ProSeek Multiplex Inflammation panel (Olink Proteomics®, Uppsala, Sweden) (see Supplementary Table S6 for a full list with names, abbreviations, detection rates and UniProt IDs). Plasma samples were measured in the Olink® testing facility in Uppsala, Sweden, where 92 matched oligonucleotide-labelled antibody pairs (probes) were incubated with the samples and allowed to pair-wise bind to the target proteins within the sample. Hybridization occurs when two probes of the same type are brought together in close proximity, which is followed by DNA polymerase extension. Finally, the corresponding DNA sequence is detected and amplified with real-time microfluidic quantitative polymerase chain reaction (qPCR) (Biomark HD Instrument, Fluidigm®, San Francisco, CA, USA)^39^.

Plasma samples were randomized on experimental plates using a randomization algorithm, which ensured randomization by age, sex and IBD subtypes (CD or UC) in order to reduce technical variation. An inter-plate intensity normalization procedure was performed prior to analysis of the data, which uses the overall median of the experiment as normalization factor. Subsequently, protein data were normalized on a log2-scale, where values were derived from inverted Ct-values of the real-time qPCR, expressed as normalized protein expression (NPX) values. NPX values represent relative quantification and constitute arbitrary units, disabling the analysis of absolute values between different proteins, and restricting it to comparisons of values for the same protein across different samples.

### Proteomics data processing

Plasma samples that deviated > 0.3 NPX from the median of internal controls were excluded from the analyses and flagged as QC-failed (*n* = 40). Eight (8) of 92 proteins (fibroblast growth factor-5 [FGF-5], interleukin-1 alpha [IL-1α], interleukin-2 [IL-2], interleukin-20 [IL-20], interleukin-22 receptor subunit alpha-1 [IL-22RA1], interleukin-33 [IL-33], leukemia inhibitory factor [LIF], and thymic stromal lymphopoietin [TSLP]) showed a very low detection rate (< 10%) and were removed for further analyses across all samples. Furthermore, tumor necrosis factor alpha (TNF-α) values were removed, as the measurement of this protein was excessively perturbed by anti-TNF-α antibodies-bound TNF-α (e.g., infliximab or adalimumab). As a consequence, the Olink TNF-α assay used for this study (ref no. 95302) delivered suboptimal results, as it employed polyclonal antibodies against TNF-α that also detect its monomeric forms, resulting in the simultaneous detection of biologically inactive forms^40,41^. Finally, proteins with NPX values below the detection limit were scored as missing values, because their inclusion in the analysis did not alter the eventual results. After data processing, a total of 83 different proteins were available for analysis for a total of 350 patients with IBD (188 CD, 162 UC).

### Statistical analysis

Baseline characteristics of the study population were presented as means ± standard deviations (SD), medians with interquartile ranges (IQR) or as proportions *n* with corresponding percentages (%). Assessment of normality was performed by visual inspection of normal probability (Q-Q) plots, histograms and kernel density plots. Differences in demographic and clinical data were compared using independent sample *t*-tests and one-way analysis of variance (ANOVA), Mann–Whitney *U*-tests or Kruskal–Wallis tests, or chi-squared tests, depending on the number of independent groups and type of variables. Patients were divided into quartiles (Q1-Q4) of fatigue scores (1–10), where plasma protein concentrations were compared using Mann-Whitney *U*-tests or Kruskal–Wallis tests. Univariable logistic regression analysis was performed to assess the association between plasma proteins and either above-/below-median fatigue scores (Q1-2 vs. Q3-4) or severe *versus* mild fatigue (Q1 vs. Q4). Multivariable backward logistic regression analysis was performed to adjust for relevant confounding variables, which were derived from univariable analysis (pre-selection threshold: *P* < 0.10). Receiver operating characteristics (ROC) statistics with the area under the curve (AUC) as overall measure of fit and corresponding 95% confidence intervals (CI) were used to assess the discriminative ability of plasma proteins with regard to the binary outcomes. ROC curves and AUCs were computed using the non-parametric, tie-corrected trapezoidal approximation method. Discriminative performance of the multivariable-adjusted models was determined by ROC estimation of the combined predicted probabilities from the models. In addition, fitted logistic regression models were internally validated using *k*-fold cross-validation (*k* = 10). In this procedure, the dataset was randomly divided into *k* equally sized folds, where each fold was then left out (10% of cases) while the model was fitted against the remaining *k*-1 folds (90% of cases, the ‘training set’) and predictions were obtained for the left-out part (the ‘test set’). This procedure was repeated ten times, where AUCs from each fold were averaged and bootstrapped to achieve statistical inference, resulting in a cross-validated AUC (cv-AUC). Statistical analysis was performed using the Python programming language (v.3.8.5, Python Software Foundation, https://www.python.org), using the *pandas* (v.1.2.3) and *sklearn* (v.0.24.1) modules and the SPSS Statistics software package (v.25.0) (SPSS Inc., Chicago, IL, USA). Data visualization was performed using *seaborn* (v.0.11.1) and *matplotlib* (v.3.4.1) packages in Python. *P*-values ≤ 0.05 were considered statistically significant.

## Supplementary Information

Supplementary Information.

## Data Availability

The datasets used and/or analysed during the current study are available from the corresponding authors on reasonable request. The data for the Groningen 1000IBD cohort can be requested at the European Genome-Phenome Archive data repository with the accession number: EGAS00001002702.
