# Supplementary material for: Proteomic analyses do not reveal subclinical inflammation in fatigued patients with clinically quiescent inflammatory bowel disease
Source: Sci Rep. 2022 Aug 26;12:14581. doi: 10.1038/s41598-022-17504-5 (PMC9418325; doi:10.1038/s41598-022-17504-5)
Supplement: Supplementary file 1 — Supplementary Information. [file 41598_2022_17504_MOESM1_ESM.docx]

**Supporting Information**

**Proteomic analyses do not reveal subclinical inflammation in fatigued patients with clinically quiescent inflammatory bowel disease**

Arno R. Bourgonje^1^, Sietse J. Wichers^1^, Shixian Hu^1,2^, Hendrik M. van Dullemen^1^, Marijn C. Visschedijk^1^, Klaas Nico Faber^1^, Eleonora A. M. Festen^1^, Gerard Dijkstra^1^, Janneke N. Samsom^3^, Rinse K. Weersma^1^, Lieke M. Spekhorst^1,4,*^

^1^Department of Gastroenterology and Hepatology, University of Groningen, University Medical Center Groningen, Groningen, the Netherlands

^2^Department of Genetics, University of Groningen, University Medical Center Groningen, Groningen, the Netherlands

^3^Department of Pediatrics, Division of Gastroenterology, Erasmus University Medical Center, Rotterdam, the Netherlands

^4^Department of Gastroenterology and Hepatology, Medisch Spectrum Twente, Enschede, the Netherlands

**Supplementary Figure S1**


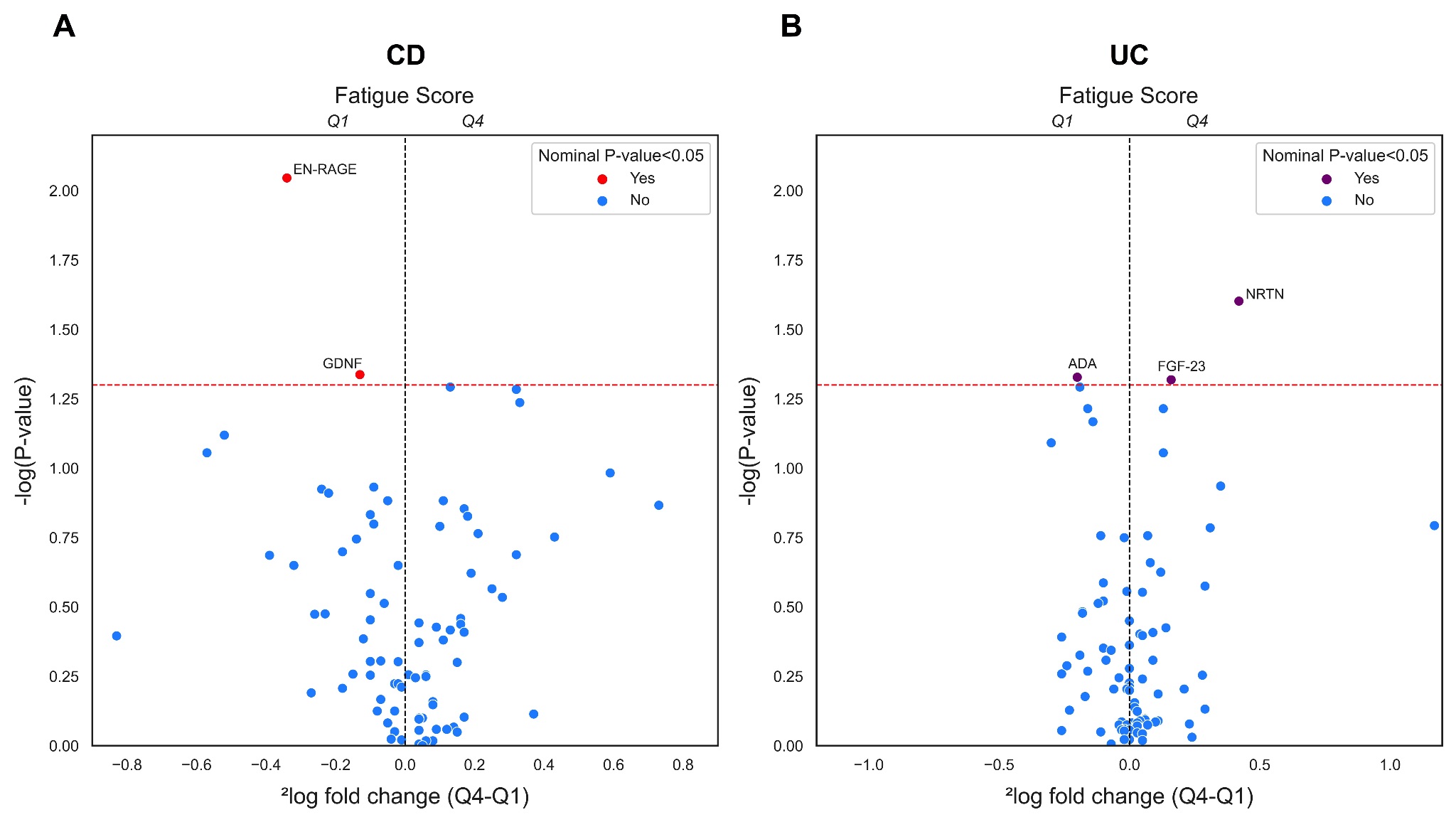


**Supplementary Figure S1**. Volcano plots demonstrating differentially abundant plasma proteins between mildly (Q1, score ranging from 0-3) fatigued patients vs. severely (Q4, score ranging from 6-10) fatigued patients with CD (A) and patients with UC (B). The red horizontal dashed lines indicate the threshold for nominal significance (nominal *P*<0.05), and the vertical black dashed lines indicate zero difference between the groups. Abbreviations: ADA, adenosine deaminase; EN-RAGE, extracellular newly identified receptor for advanced glycation end-product binding protein; GDNF, glial cell line-derived neurotrophic factor; FGF-23, fibroblast growth factor-23; NRTN, neurturin; Q1, first quartile; Q4, fourth quartile.

**Supplementary Figure S2**


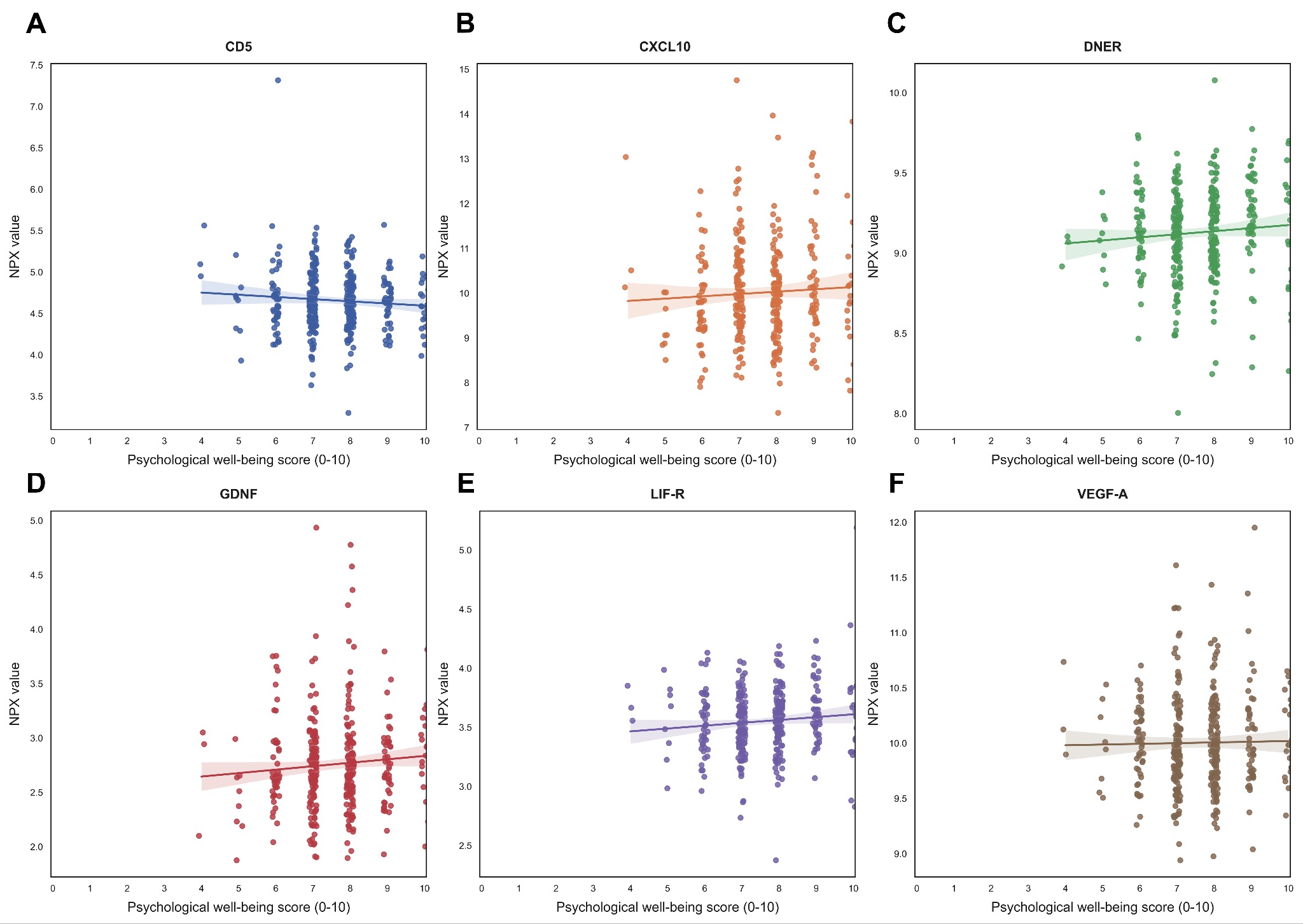


**Supplementary Figure S2**. Associations between the top six (6) differentially abundant plasma proteins between mildly fatigued (Q1) patients and severely fatigued (Q4) patients and psychological well-being scores. Abbreviations: CD5, T-cell surface glycoprotein CD5; CXCL10, C-X-C motif chemokine ligand 10; DNER, Delta and Notch-like epidermal growth factor-related receptor; GDNF, glial cell line-derived neurotrophic factor; leukemia inhibitory factor receptor; NPX, normalized protein expression; vascular endothelial growth factor A (VEGF-A).

**Supplementary Figure S3**


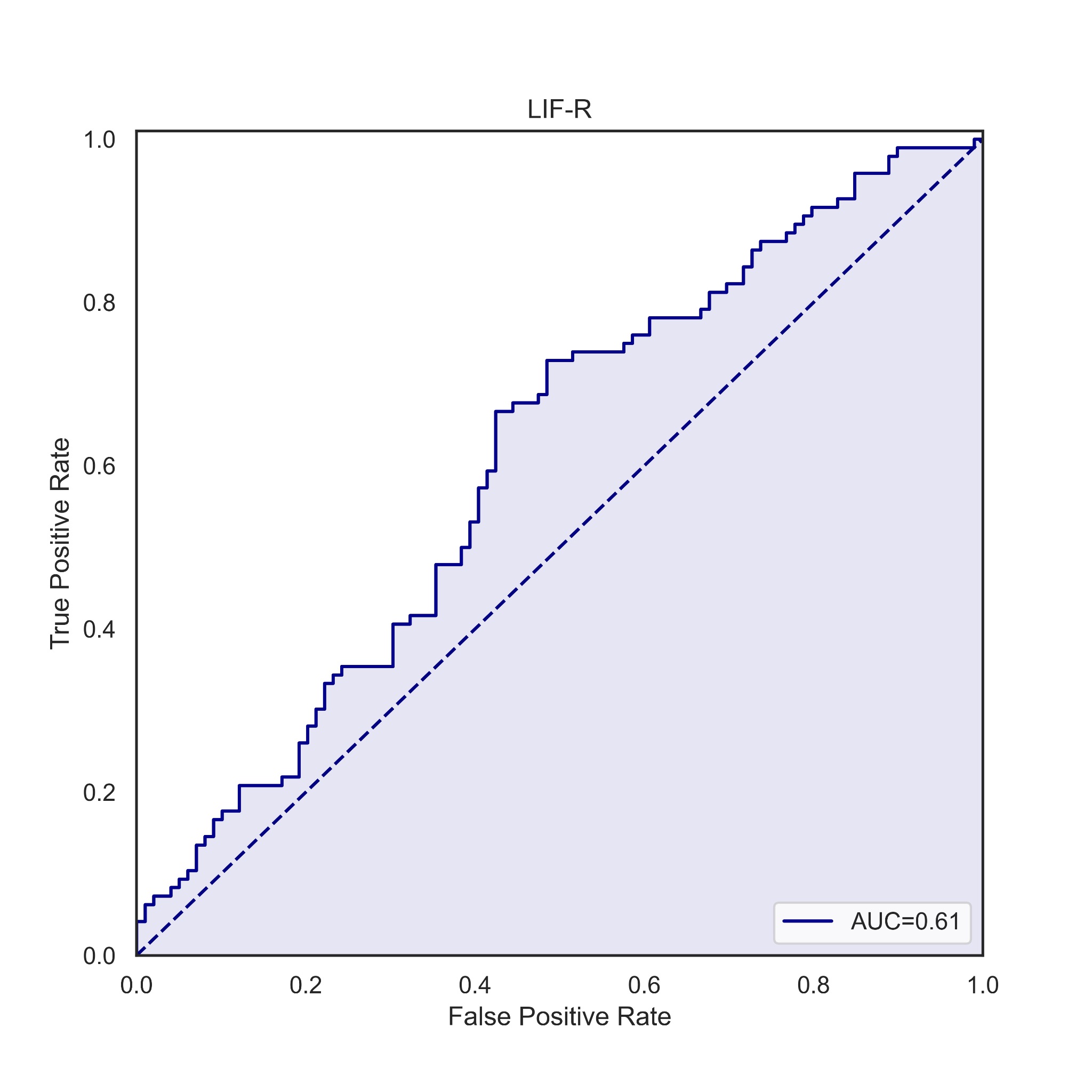


**Supplementary Figure S3**. Receiver operating characteristics (ROC) curve demonstrating the discriminative performance of LIF-R with regard to severely fatigued (Q4) vs. mildly fatigued (Q1) patients with IBD. Abbreviations: AUC, area under the curve; LIF-R, leukemia inhibitory factor receptor.

**Supplementary Figure S4**


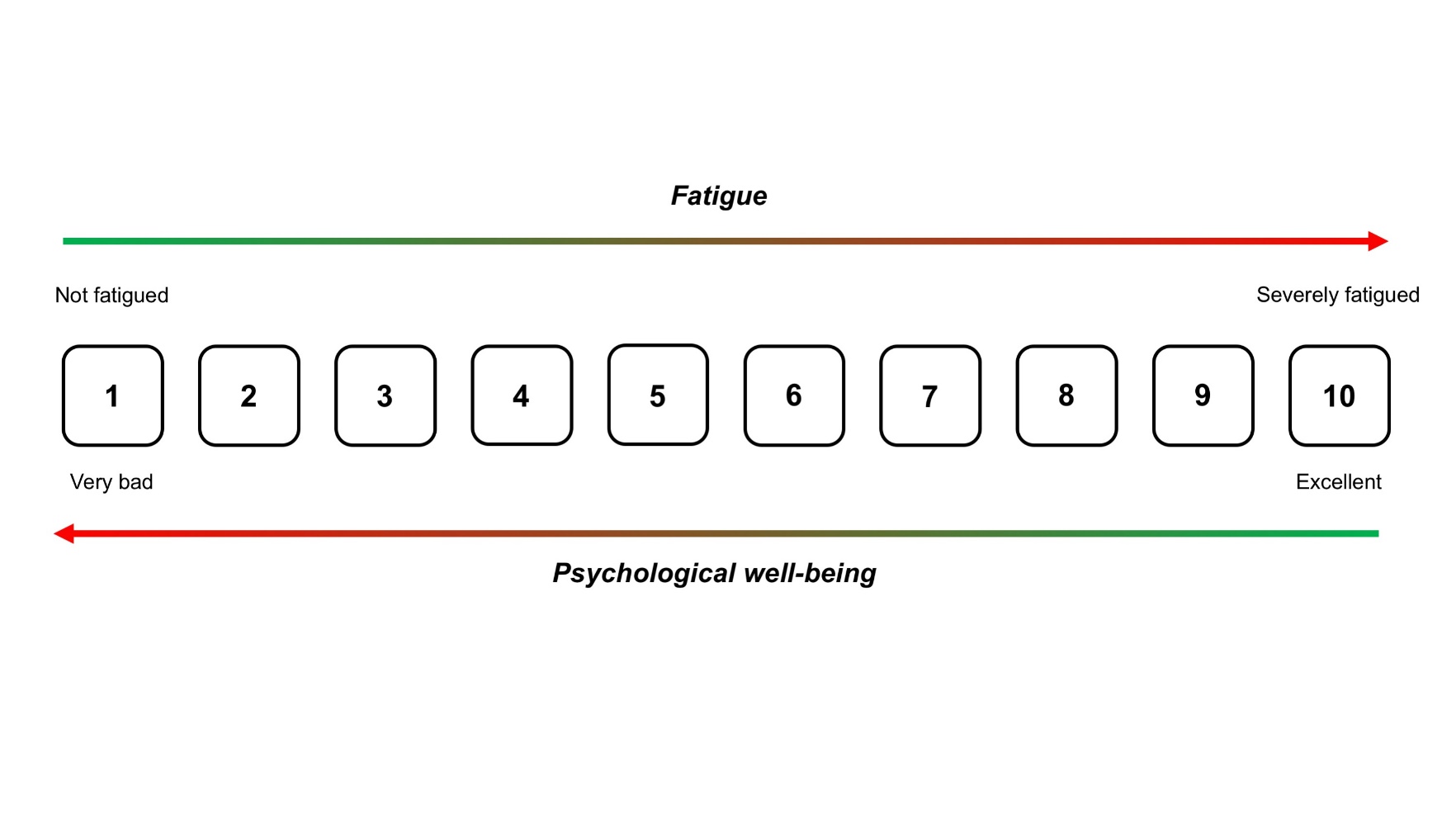


**Supplementary Figure S4**. Assessment of fatigue and psychological well-being on a visual analogue (VAS) or 10-point Likert scale. Fatigue scores ranged from 1 (being not fatigued at all) to 10 (being severely fatigued) and psychological well-being scores also from 1 (feeling very bad) to 10 (feeling excellent).

**Supplementary Table S1**. Demographic and clinical characteristics of the study population, for the total cohort and stratified by below- and above-median fatigue scores.

| **Variable** | **Total** | **Below-median fatigue (0-4)** | **Above-median fatigue (5-10)** | ***P*-value** |
| --- | --- | --- | --- | --- |
|  | *n* = 350 | *n* = 176 | *n* = 174 |  |
| Age (years) | 41.5 ± 15.0 | 42.4 ± 15.2 | 40.7 ± 14.8 | 0.30 |
| Sex, *n* (%) |  |  |  | <0.01 |
| Male | 179 (51.1) | 108 (61.4) | 71 (40.8) |  |
| Female | 171 (48.9) | 68 (38.6) | 103 (59.2) |  |
| BMI (kg/m^2^) | 24.5 [22.3;27.5] | 24.4 [22.4;27.3] | 24.6 [22.2;27.8] | 0.73 |
| IBD diagnosis, *n* (%) |  |  |  | 0.02 |
| CD | 188 (53.7) | 84 (47.7) | 104 (59.8) |  |
| UC | 162 (46.3) | 92 (52.3) | 70 (40.2) |  |
| Current smoking, *n* (%) | 334 (95.4) | 166 (94.3) | 168 (96.6) | <0.01 |
| Yes | 65 (18.6) | 21 (12.7) | 44 (26.2) |  |
| No | 269 (76.9) | 145 (87.3) | 124 (73.8) |  |
| **Montreal classification** |  |  |  |  |
| Montreal Age (A), *n* (%) | 349 (99.7) | 176 (100) | 173 (98.3) | 0.57 |
| A1 (≤16 years) | 52 (14.9) | 27 (15.3) | 25 (14.5) |  |
| A2 (17-40 years) | 223 (63.7) | 108 (61.4) | 115 (66.5) |  |
| A3 (>40 years) | 74 (21.1) | 41 (23.3) | 33 (19.1) |  |
| Montreal Location (L), *n* (%) | 188 (100) | 84 (100) | 104 (100) | 0.06 |
| L1 (ileal disease) | 63 (33.5) | 26 (31.0) | 37 (35.6) |  |
| L2 (colonic disease) | 46 (24.5) | 27 (32.1) | 19 (18.3) |  |
| L3 (ileocolonic disease) | 63 (33.5) | 22 (26.2) | 41 (39.4) |  |
| L4 (upper GI disease) | 5 (2.7) | 2 (2.4) | 3 (2.9) |  |
| L1 + L4 | 4 (2.1) | 1 (1.2) | 3 (2.9) |  |
| L2 + L4 | 4 (2.1) | 3 (3.6) | 1 (1.0) |  |
| L3 + L4 | 3 (1.6) | 3 (3.6) | 0 (0.0) |  |
| Montreal Behavior (B), *n* (%) | 188 (100) | 84 (100) | 104 (100) | 0.24 |
| B1 (non-stricturing, non-penetrating) | 88 (46.8) | 32 (38.1) | 56 (53.8) |  |
| B2 (stricturing) | 32 (17.0) | 14 (16.7) | 18 (17.3) |  |
| B3 (penetrating) | 16 (8.5) | 10 (11.9) | 6 (5.8) |  |
| B1 + P (perianal disease) | 17 (9.0) | 8 (9.5) | 9 (8.7) |  |
| B2 + P (perianal disease) | 18 (9.6) | 10 (11.9) | 8 (7.7) |  |
| B3 + P (perianal disease) | 17 (9.0) | 10 (11.9) | 7 (6.7) |  |
| Montreal Extension (E), *n* (%) | 161 (99.4) | 92 (100) | 69 (98.6) | 0.48 |
| E1 (proctitis) | 29 (17.9) | 14 (15.2) | 15 (21.7) |  |
| E2 (left-sided colitis) | 53 (32.7) | 33 (35.9) | 20 (29.0) |  |
| E3 (pancolitis) | 79 (48.8) | 45 (48.9) | 34 (49.3) |  |
| **Medication use** |  |  |  |  |
| Aminosalicylates, *n* (%) | 130 (37.1) | 69 (39.2) | 61 (35.1) | 0.42 |
| Thiopurines, *n* (%) | 138 (39.4) | 62 (35.2) | 76 (43.7) | 0.11 |
| Steroids, *n* (%) | 52 (14.9) | 31 (17.6) | 21 (12.1) | 0.15 |
| Calcineurin inhibitors, *n* (%) | 11 (3.1) | 7 (4.0) | 4 (2.3) | 0.37 |
| Methotrexate, *n* (%) | 16 (4.6) | 6 (3.4) | 10 (5.7) | 0.30 |
| Mycophenolate mofetil, *n* (%) | 7 (2.0) | 4 (2.3) | 3 (1.7) | 0.71 |
| TNF-α-antagonists, *n* (%)^†^ | 53 (15.1) | 22 (12.5) | 31 (17.8) | 0.17 |
| **Surgical history** |  |  |  |  |
| Ileocecal resection, *n* (%) | 52 (14.9) | 23 (13.1) | 29 (16.7) | 0.34 |
| Colon resection (or partial), *n* (%) | 49 (14.0) | 22 (12.5) | 27 (15.5) | 0.42 |
| **Laboratory parameters** |  |  |  |  |
| Hb (mmol/L) (males) | 9.4 [9.0-9.8] | 9.4 [9.1-9.8] | 9.4 [9.0-9.8] | 0.88 |
| Hb (mmol/L) (females) | 8.2 [7.9-8.7] | 8.4 [7.9-8.8] | 8.2 [7.9-8.6] | 0.24 |
| CRP (mg/L)^*^ | 5.0 [1.4-5.0] | 4.6 [1.1-5.0] | 5.0 [1.8-5.0] | 0.10 |

Data are presented as proportions *n* with corresponding percentages (%) or as median [interquartile range, IQR] in case of continuous variables. *P*-values ≤0.05 were considered statistically significant. ^†^The use of TNF-α-antagonists included use of the following compounds: infliximab, adalimumab, golimumab and certolizumab pegol. Abbreviations: BMI, body-mass index; CD, Crohn’s disease; IBD, inflammatory bowel disease; TNF-α, tumor necrosis factor alpha; UC, ulcerative colitis. ^*^Numericized lower limits of detection (<5 mg/L) were included in calculating median and IQR, but may falsely represent the (unknown) true biological value. All CRP values were < 5 mg/L, as this was one of the study’s inclusion criteria.

**Supplementary Table S2**. Plasma protein concentrations (shown as normalized protein expression [NPX] values) compared between patients with the lowest fatigue scores (Q1) and those with the highest fatigue scores (Q4).

| **Protein** | **Total** | **Q1 fatigue score (0-3)** | **Q4 fatigue score (6-10)** | **Δ Q4-Q1^†^** | ***P*-value^*^** |
| --- | --- | --- | --- | --- | --- |
|  | *n*=195 | *n*=96 | *n*=99 |  |  |
| LIF-R | 3.56 [3.39-3.70] | 3.60 [3.46-3.73] | 3.48 [3.36-3.67] | -0.12 | 0.009 |
| VEGF-A | 9.98 [9.73-10.3] | 9.91 [9.67-10.2] | 10.1 [9.85-10.3] | 0.17 | 0.016 |
| DNER | 9.15 [8.99-9.38] | 9.23 [8.99-9.39] | 9.10 [8.97-9.31] | -0.13 | 0.034 |
| CD5 | 4.66 [4.41-4.87] | 4.61 [4.35-4.85] | 4.70 [4.47-4.96] | 0.09 | 0.035 |
| GDNF | 2.68 [2.47-2.96] | 2.74 [2.54-3.01] | 2.64 [2.38-2.95] | -0.10 | 0.037 |
| CXCL10 | 9.81 [9.32-10.4] | 9.96 [9.46-10.6] | 9.68 [9.18-10.2] | -0.28 | 0.044 |
| EN-RAGE | 2.49 [2.15-2.87] | 2.60 [2.20-2.97] | 2.40 [2.10-2.81] | -0.20 | 0.053 |
| IL-20RA | 1.67 [1.51-1.79] | 1.63 [1.51-1.72] | 1.71 [1.52-1.89] | 0.08 | 0.059 |
| TNFSF14 | 4.33 [3.91-4.65] | 4.23 [3.80-4.56] | 4.40 [4.04-4.68] | 0.17 | 0.069 |
| CASP-8 | 3.25 [2.76-3.69] | 3.38 [2.82-3.85] | 3.21 [2.75-3.53] | -0.17 | 0.085 |
| CD40 | 12.0 [11.6-12.3] | 11.9 [11.6-12.2] | 12.0 [11.6-12.4] | 0.16 | 0.092 |
| TRANCE | 4.88 [4.47-5.29] | 4.96 [4.47-5.42] | 4.84 [4.46-5.18] | -0.12 | 0.110 |
| IL-18 | 8.55 [8.22-8.92] | 8.66 [8.28-8.96] | 8.50 [8.17-8.87] | -0.16 | 0.112 |
| CXCL9 | 7.24 [6.60-7.85] | 7.37 [6.90-7.90] | 7.03 [7.49-7.85] | -0.34 | 0.121 |
| CXCL1 | 10.8 [10.3-11.3] | 10.7 [10.3-11.2] | 10.9 [10.4-11.4] | 0.17 | 0.130 |
| FGF-21 | 4.75 [3.77-5.73] | 4.70 [3.66-5.47] | 5.01 [3.98 [5.84] | 0.31 | 0.137 |
| IL-10RA | 1.18 [1.00-1.74] | 1.12 [0.96-1.70] | 1.24 [1.01-2.09] | 0.12 | 0.139 |
| OPG | 10.1 [9.8-10.4] | 10.1 [9.82-10.3] | 10.1 [9.86-10.5] | 0.05 | 0.140 |
| SIRT2 | 7.27 [6.58-8.09] | 7.11 [6.52-7.89] | 7.57 [6.65-8.25] | 0.46 | 0.154 |
| CXCL5 | 11.5 [10.7-12.4] | 11.4 [10.7-12.2] | 11.6 [10.8-12.5] | 0.21 | 0.157 |
| AXIN1 | 6.21 [5.41-7.13] | 6.10 [5.36-6.96] | 6.39 [5.42-7.21] | 0.29 | 0.163 |
| β-NGF | 1.47 [1.36-1.62] | 1.50 [1.37-1.67] | 1.44 [1.36-1.59] | -0.06 | 0.167 |
| NRTN | 1.74 [1.50-2.59] | 1.57 [1.39-2.42] | 1.91 [1.59-2.84] | 0.34 | 0.188 |
| 4E-BP1 | 9.00 [8.37-9.60] | 8.96 [8.30-9.52] | 9.09 [8.45-9.68] | 0.13 | 0.202 |
| STAMBP | 7.34 [6.72-8.09] | 7.25 [6.64-8.03] | 7.47 [6.75-8.28] | 0.22 | 0.236 |
| CD8A | 10.1 [9.63-10.6] | 10.1 [9.69-10.7] | 10.0 [9.51-10.6] | -0.08 | 0.245 |
| MCP-2 | 9.28 [8.93-9.61] | 9.25 [8.86-9.58] | 9.34 [8.95-9.71] | 0.09 | 0.265 |
| CCL11 | 7.28 [6.91-7.65] | 7.35 [6.97-7.67] | 7.23 [6.85-7.64] | -0.12 | 0.266 |
| SCF | 9.68 [9.39-9.87] | 9.70 [9.45-9.91] | 9.64 [9.38-9.85] | -0.06 | 0.273 |
| MCP-3 | 1.93 [1.70-2.14] | 1.89 [1.63-2.10] | 1.93 [1.73-2.17] | 0.04 | 0.289 |
| IL-7 | 3.31 [2.90-3.61] | 3.24 [2.89-3.59] | 3.35 [2.95-3.79] | 0.11 | 0.304 |
| CSF-1 | 10.3 [10.2-10.5] | 10.3 [10.1-10.4] | 10.3 [10.2-10.5] | 0.04 | 0.318 |
| IL-10RB | 6.09 [5.91-6.26] | 6.06 [5.86-6.22] | 6.09 [5.95-6.27] | 0.03 | 0.322 |
| CD244 | 7.17 [6.90-7.54] | 7.09 [6.89-7.45] | 7.19 [6.93-7.58] | 0.10 | 0.325 |
| TWEAK | 9.69 [9.47-9.91] | 9.75 [9.53-9.92] | 9.66 [9.40-9.90] | -0.09 | 0.330 |
| FGF-23 | 3.37 [3.17-3.62] | 3.33 [3.16-3.60] | 3.39 [3.17-3.72] | 0.06 | 0.375 |
| IL-4 | 1.70 [1.34-2.58] | 1.78 [1.39-2.76] | 1.63 [1.29-2.31] | -0.15 | 0.394 |
| IL-12B | 6.38 [5.91-6.89] | 6.34 [5.91-6.76] | 6.41 [5.93-6.96] | 0.07 | 0.399 |
| IL-24 | 2.09 [1.84-2.65] | 2.06 [1.83-2.52] | 2.13 [1.87-3.14] | 0.07 | 0.426 |
| TNFRSF9 | 6.64 [6.37-6.95] | 6.68 [6.41-6.94] | 6.61 [6.29-6.97] | -0.07 | 0.436 |
| IL-10 | 4.06 [3.67-4.52] | 4.19 [3.64-4.73] | 4.05 [3.67-4.51] | -0.14 | 0.492 |
| CD6 | 5.21 [4.93-5.57] | 5.19 [4.92-5.50] | 5.22 [4.98-5.61] | 0.03 | 0.521 |
| MCP-1 | 10.9 [10.6-11.1] | 10.9 [10.6-11.2] | 10.9 [10.6-11.1] | -0.01 | 0.522 |
| MCP-4 | 13.6 [13.3-14.1] | 13.6 [13.2-14.2] | 13.7 [13.3-14.1] | 0.12 | 0.532 |
| IL-17C | 1.87 [1.73-2.11] | 1.87 [1.74-2.11] | 1.89 [1.70-2.18] | 0.02 | 0.536 |
| uPA | 9.71 [9.49-9.93] | 9.71 [9.56-9.93] | 9.72 [9.45-9.93] | 0.01 | 0.539 |
| CCL4 | 5.96 [5.54-6.34] | 6.03 [5.62-6.30] | 5.84 [5.54-6.36] | -0.19 | 0.556 |
| IL-5 | 2.32 [1.84-3.26] | 2.35 [1.89-3.88] | 2.28 [1.80-3.05] | -0.07 | 0.559 |
| LAP TGF-β-1 | 7.55 [7.24-7.86] | 7.54 [7.25-7.81] | 7.58 [7.22-7.94] | 0.04 | 0.580 |
| TNFB | 4.85 [4.53-5.07] | 4.87 [4.55-5.07] | 4.82 [4.48-5.06] | -0.05 | 0.589 |
| ARTN | 1.40 [1.18-1.94] | 1.35 [1.16-2.08] | 1.52 [1.33-1.82] | 0.17 | 0.591 |
| SLAMF1 | 3.07 [2.76-3.37] | 3.13 [2.74-3.43] | 3.03 [2.77-3.34] | -0.10 | 0.605 |
| CST5 | 5.90 [5.62-6.21] | 5.92 [5.60-6.27] | 5.89 [5.64-6.21] | -0.03 | 0.631 |
| OSM | 4.62 [4.00-5.31] | 4.58 [3.84-5.31] | 4.66 [4.08-5.28] | 0.08 | 0.669 |
| CCL23 | 9.95 [9.65-10.2] | 9.92 [9.65-10.2] | 9.96 [9.67-10.2] | 0.04 | 0.675 |
| IL-15RA | 0.98 [0.86-1.13] | 0.99 [0.86-1.13] | 0.97 [0.85-1.13] | -0.02 | 0.683 |
| CCL25 | 5.96 [5.50-6.37] | 5.93 [5.51-6.32] | 5.99 [5.44-6.38] | 0.06 | 0.743 |
| CCL20 | 6.83 [6.45-7.37] | 6.77 [6.49-7.36] | 6.86 [6.38-7.38] | 0.09 | 0.751 |
| MMP-1 | 10.1 [9.39-10.9] | 10.1 [9.40-11.0] | 10.0 [9.39-10.9] | -0.04 | 0.763 |
| CX3CL1 | 6.53 [6.25-6.82] | 6.51 [6.25-6.79] | 6.55 [6.25-6.89] | 0.04 | 0.764 |
| IL-17A | 1.94 [1.65-2.25] | 1.94 [1.62-2.25] | 1.96 [1.67-2.30] | 0.02 | 0.765 |
| Flt3L | 9.34 [8.93-9.62] | 9.36 [8.97-9.65] | 9.32 [8.92-9.61] | -0.04 | 0.768 |
| FGF-19 | 8.00 [7.04-8.62] | 8.14 [6.80-8.61] | 7.94 [7.14-8.63] | -0.20 | 0.774 |
| CCL28 | 2.40 [2.24-2.68] | 2.45 [2.21-2.68] | 2.38 [2.25-2.68] | -0.07 | 0.776 |
| HGF | 8.80 [8.58-9.13] | 8.80 [8.61-9.16] | 8.80 [8.54-9.12] | 0.00 | 0.780 |
| IFN-γ | 6.33 [5.53-7.25] | 6.36 [5.54-7.29] | 6.28 [5.50-7.21] | -0.06 | 0.782 |
| TRAIL | 8.69 [8.47-8.93] | 8.68 [8.49-8.90] | 8.73 [8.45-8.94] | 0.05 | 0.811 |
| CXCL11 | 8.50 [7.94-9.13] | 8.46 [7.96-9.03] | 8.58 [7.93-9.13] | 0.12 | 0.813 |
| IL-13 | 2.34 [1.78-3.58] | 2.30 [1.78-3.64] | 2.67 [1.76-3.73] | 0.37 | 0.817 |
| CXCL6 | 8.82 [8.33-9.41] | 8.82 [8.34-9.36] | 8.82 [8.23-9.47] | 0.00 | 0.818 |
| CCL3 | 5.45 [5.11-5.85] | 5.42 [5.14-5.83] | 5.46 [5.10-5.89] | 0.04 | 0.829 |
| IL-2RB | 1.86 [1.71-2.22] | 1.91 [1.73-2.17] | 1.82 [1.71-2.40] | -0.09 | 0.840 |
| CDCP1 | 3.39 [3.08-3.93] | 3.41 [3.07-3.90] | 3.38 [3.10-3.97] | -0.03 | 0.842 |
| PD-L1 | 7.03 [6.74-7.25] | 7.04 [6.69-7.27] | 7.02 [6.77-7.22] | -0.02 | 0.879 |
| TGF-α | 4.15 [3.98-4.33] | 4.14 [3.96-4.34] | 4.15 [4.01-4.33] | 0.01 | 0.881 |
| MMP-10 | 6.61 [6.29-6.99] | 6.61 [6.33-6.98] | 6.68 [6.22-7.00] | 0.07 | 0.887 |
| ST1A1 | 4.13 [3.56-4.55] | 4.12 [3.48-4.61] | 4.13 [3.61 [4.55] | 0.01 | 0.921 |
| ADA | 4.56 [4.30-4.79] | 4.56 [4.28-4.80] | 4.56 [4.33-4.79] | 0.00 | 0.957 |
| IL-18R1 | 8.40 [8.03-8.62] | 8.39 [7.97-8.63] | 8.40 [8.07-8.61] | 0.01 | 0.967 |
| IL-6 | 3.32 [2.82-3.74] | 3.35 [2.82-3.70] | 3.30 [2.82-3.77] | -0.05 | 0.968 |
| CCL19 | 9.68 [9.29-10.1] | 9.65 [9.29-10.1] | 9.73 [9.28-10.1] | 0.08 | 0.973 |
| NT-3 | 3.23 [2.98-3.54] | 3.22 [3.02-3.53] | 3.24 [2.96-3.55] | 0.02 | 0.988 |
| IL-8 | 5.65 [5.28-6.04] | 5.64 [5.32-6.06] | 5.68 [5.25-6.01] | 0.04 | 0.999 |

Data are presented as medians with interquartile ranges [IQR]. *P*-values ≤0.05 were considered to be nominally statistically significant.^†^ Difference in protein concentration (NPX) between patients with highest fatigue scores (Q4) and with the lowest fatigue scores (Q1).^*^Nominal *P*-values, which all did not reach statistical significance after adjusting for multiple comparisons (using the Benjamini-Hochberg procedure, all proteins having a false discovery rate [FDR]>0.1).

**Supplementary Table S3**. Plasma protein concentrations (shown as normalized protein expression [NPX] values) compared between patients with below- and above-median fatigue scores.

| **Protein** | **Total cohort** | **Below-median fatigue (0-4)** | **Above-median fatigue (5-10)** | **Δ Median^†^** | ***P*-value^*^** |
| --- | --- | --- | --- | --- | --- |
|  | *n*=350 | *n*=176 | *n*=174 |  |  |
| LIF-R | 3.54 [3.38-3.72] | 3.59 [3.40-3.74] | 3.51 [3.36-3.68] | -0.08 | 0.021 |
| DNER | 9.14 [8.97-9.32] | 9.20 [8.97-8.34] | 9.10 [8.97-9.26] | -0.10 | 0.022 |
| CXCL10 | 9.91 [9.33-10.6] | 10.0 [9.46-10.7] | 9.80 [9.20-10.4] | -0.20 | 0.028 |
| GDNF | 2.69 [2.49-2.96] | 2.75 [2.55-2.97] | 2.65 [2.42-2.95] | -0.10 | 0.030 |
| EN-RAGE | 2.49 [2.17-2.89] | 2.59 [2.20-2.94] | 2.41 [2.12-2.85] | -0.18 | 0.045 |
| CCL11 | 7.25 [6.93-7.65] | 7.33 [6.97-7.68] | 7.20 [6.83-7.62] | -0.13 | 0.063 |
| β-NGF | 1.47 [1.37-1.64] | 1.50 [1.39-1.67] | 1.44 [1.36-1.61] | -0.06 | 0.072 |
| MCP-3 | 1.92 [1.70-2.17] | 1.88 [1.64-2.10] | 1.93 [1.75-2.23] | 0.05 | 0.075 |
| CXCL9 | 7.29 [6.63-7.87] | 7.39 [6.84-7.91] | 7.16 [6.53-7.82] | -0.23 | 0.081 |
| NRTN | 1.77 [1.50-2.61] | 1.57 [1.38-2.42] | 1.95 [1.59-2.78] | 0.38 | 0.084 |
| IL-10RA | 1.18 [0.98-1.68] | 1.14 [0.96-1.60] | 1.21 [1.01-1.80] | 0.07 | 0.088 |
| TRANCE | 4.93 [4.48-5.32] | 4.97 [4.49-5.43] | 4.89 [4.47-5.22] | -0.08 | 0.088 |
| CSF-1 | 10.3 [10.2-10.4] | 10.3 [10.1-10.4] | 10.3 [10.2-10.5] | 0.05 | 0.094 |
| MCP-2 | 9.28 [8.89-9.64] | 9.24 [8.79-9.59] | 9.34 [8.98-9.71] | 0.10 | 0.098 |
| MMP-1 | 10.1 [9.46-10.8] | 10.3 [9.60-10.9] | 10.0 [9.36-10.8] | -0.26 | 0.121 |
| uPA | 9.73 [9.48-9.92] | 9.74 [9.56-9.93] | 9.71 [9.41-9.92] | -0.03 | 0.130 |
| CD8A | 10.1 [9.63-10.6] | 10.1 [9.68-10.6] | 10.0 [9.54-10.6] | -0.08 | 0.150 |
| TNFSF14 | 4.28 [3.89-4.65] | 4.24 [3.83-4.60] | 4.34 [3.97-4.68] | 0.10 | 0.161 |
| HGF | 8.80 [8.55-9.13] | 8.82 [8.58-9.17] | 8.78 [8.51-9.10] | -0.04 | 0.190 |
| SIRT2 | 7.25 [6.57-8.00] | 7.08 [6.52-7.87] | 7.40 [6.58-8.10] | 0.32 | 0.195 |
| CXCL5 | 11.5 [10.8-12.3] | 11.5 [10.7-12.3] | 11.6 [10.9-12.4] | 0.15 | 0.196 |
| VEGF-A | 9.94 [9.70-10.3] | 9.92 [9.69-10.3] | 9.99 [9.72-10.3] | 0.07 | 0.211 |
| SLAMF1 | 3.06 [2.76-3.39] | 3.13 [2.78-3.43] | 3.02 [2.75-3.35] | -0.11 | 0.212 |
| CD5 | 4.65 [4.43-4.86] | 4.64 [4.38-4.85] | 4.67 [4.47-4.89] | 0.03 | 0.221 |
| TNFRSF9 | 6.63 [6.32-6.94] | 6.65 [6.37-6.94] | 6.59 [6.27-6.94] | -0.06 | 0.221 |
| IL-18 | 8.52 [8.17-8.88] | 8.55 [8.24-8.91] | 8.50 [8.13-8.87] | -0.05 | 0.222 |
| IL-17C | 1.83 [1.70-2.10] | 1.81 [1.71-2.08] | 1.90 [1.70-2.11] | 0.09 | 0.240 |
| IL-20RA | 1.66 [1.51-1.88] | 1.63 [1.51-1.79] | 1.67 [1.51-1.90] | 0.04 | 0.242 |
| TNFB | 4.82 [4.53-5.07] | 4.87 [4.54-5.07] | 4.80 [4.47-5.05] | -0.07 | 0.242 |
| MCP-1 | 10.9 [10.6-11.1] | 10.9 [10.7-11.2] | 10.8 [10.6-11.1] | -0.07 | 0.255 |
| IL-15RA | 0.96 [0.85-1.11] | 0.98 [0.86-1.12] | 0.96 [0.83-1.11] | -0.02 | 0.261 |
| 4E-BP1 | 8.96 [8.33-9.49] | 8.92 [8.32-9.44] | 9.00 [8.35-9.59] | 0.08 | 0.285 |
| CASP-8 | 3.21 [2.74-3.69] | 3.22 [2.75-3.78] | 3.21 [2.72-3.59] | -0.01 | 0.312 |
| Flt3L | 9.32 [8.93-9.65] | 9.36 [8.99-9.67] | 9.26 [8.90-9.61] | -0.10 | 0.335 |
| TGF-α | 4.14 [3.98-4.33] | 4.15 [3.98-4.34] | 4.14 [3.98-4.32] | -0.01 | 0.356 |
| CD40 | 12.0 [11.6-12.3] | 11.9 [11.6-12.2] | 12.0 [11.6-12.4] | 0.10 | 0.374 |
| PD-L1 | 7.02 [6.71-7.28] | 7.04 [6.69-7.33] | 6.99 [6.72-7.23] | -0.05 | 0.392 |
| IL-5 | 2.43 [1.90-3.37] | 2.50 [1.99-3.33] | 2.36 [1.84-3.39] | -0.14 | 0.393 |
| TWEAK | 9.69 [9.47-9.91] | 9.72 [9.52-9.91] | 9.66 [9.43-9.96] | -0.06 | 0.398 |
| CCL20 | 6.80 [6.37-7.37] | 6.76 [6.35-7.34] | 6.85 [6.37-7.43] | 0.09 | 0.404 |
| CCL25 | 5.91 [5.49-6.32] | 5.91 [5.52-6.37] | 5.86 [5.42-6.32] | -0.05 | 0.431 |
| AXIN1 | 6.17 [5.35-7.12] | 6.15 [5.36-6.96] | 6.21 [5.33-7.21] | 0.06 | 0.454 |
| IFN-γ | 6.42 [5.66-7.39] | 6.42 [5.74-7.46] | 6.40 [5.56-7.29] | -0.02 | 0.454 |
| IL-10 | 4.07 [3.75-4.51] | 4.07 [3.74-4.56] | 4.06 [3.77-4.51] | -0.01 | 0.460 |
| STAMBP | 7.30 [6.66-8.08] | 7.22 [6.64-7.99] | 7.36 [6.66-8.19] | 0.14 | 0.475 |
| TRAIL | 8.69 [8.45-8.90] | 8.71 [8.48-8.89] | 8.67 [8.40-8.93] | -0.04 | 0.477 |
| IL-7 | 3.28 [2.85-3.63] | 3.24 [2.88-3.60] | 3.32 [2.82-3.74] | 0.08 | 0.480 |
| OPG | 10.1 [9.83-10.4] | 10.1 [9.83-10.3] | 10.1 [9.84-10.4] | 0.01 | 0.486 |
| CXCL1 | 10.8 [10.2-11.3] | 10.8 [10.2-11.2] | 10.8 [10.2-11.4] | 0.00 | 0.492 |
| IL-12B | 6.41 [5.96-6.90] | 6.38 [5.97-6.79] | 6.43 [5.94-6.97] | 0.05 | 0.517 |
| CD6 | 5.22 [4.92-5.57] | 5.26 [4.94-5.55] | 5.21 [4.91-5.60] | -0.05 | 0.532 |
| SCF | 9.66 [9.42-9.87] | 9.66 [9.42-9.89] | 9.64 [9.42-9.86] | -0.02 | 0.550 |
| IL-2RB | 1.87 [1.71-2.33] | 1.91 [1.71-2.22] | 1.85 [1.72-2.46] | -0.06 | 0.552 |
| CCL28 | 2.44 [2.22-2.71] | 2.47 [2.21-2.70] | 2.40 [2.22-2.71] | -0.07 | 0.565 |
| IL-24 | 2.09 [1.87-2.67] | 2.11 [1.87-2.56] | 2.05 [1.86-2.73] | -0.06 | 0.636 |
| IL-13 | 2.22 [1.82-3.34] | 2.27 [1.80-3.23] | 2.20 [1.86-3.63] | -0.07 | 0.638 |
| LAP TGF-β-1 | 7.53 [7.19-7.86] | 7.52 [7.19-7.83] | 7.53 [7.18-7.91] | 0.01 | 0.645 |
| CXCL6 | 8.82 [8.30-9.40] | 8.82 [8.37-9.35] | 8.81 [8.18-9.43] | -0.01 | 0.672 |
| IL-18R1 | 8.35 [8.00-8.59] | 8.32 [7.96-8.62] | 8.38 [8.05-8.58] | 0.06 | 0.673 |
| OSM | 4.66 [3.97-5.27] | 4.61 [3.88-5.30] | 4.68 [4.05-5.25] | 0.07 | 0.685 |
| CD244 | 7.14 [6.87-7.52] | 7.12 [6.89-7.47] | 7.18 [6.82-7.55] | 0.06 | 0.697 |
| IL-8 | 5.64 [5.25-6.00] | 5.63 [5.30-6.02] | 5.66 [5.19-5.97] | 0.03 | 0.700 |
| CCL3 | 5.46 [5.08-5.87] | 5.46 [5.08-5.86] | 5.46 [5.08-5.89] | 0.00 | 0.708 |
| CST5 | 5.87 [5.59-6.16] | 5.88 [5.59-6.19] | 5.86 [5.60-6.14] | -0.02 | 0.716 |
| NT-3 | 3.23 [2.96-3.50] | 3.23 [2.96-3.51] | 3.24 [2.95-3.47] | 0.01 | 0.716 |
| MMP-10 | 6.61 [6.22-6.99] | 6.59 [6.27-6.98] | 6.63 [6.21-7.01] | 0.04 | 0.717 |
| CCL23 | 9.92 [9.65-10.2] | 9.91 [9.64-10.2] | 9.94 [9.65-10.2] | 0.03 | 0.745 |
| CCL4 | 5.89 [5.48-6.33] | 5.95 [5.46-6.30] | 5.87 [5.51-6.34] | -0.08 | 0.749 |
| CDCP1 | 3.43 [3.06-3.90] | 3.45 [3.07-3.90] | 3.38 [3.05-3.90] | -0.07 | 0.749 |
| IL-4 | 1.74 [1.26-2.90] | 1.78 [1.24-3.32] | 1.69 [1.27-2.76] | -0.09 | 0.749 |
| FGF-23 | 3.37 [3.16-3.63] | 3.35 [3.16-3.63] | 3.38 [3.16-3.65] | 0.03 | 0.756 |
| CXCL11 | 8.46 [7.95-9.17] | 8.44 [8.01-9.03] | 8.55 [7.89-9.36] | 0.11 | 0.758 |
| FGF-21 | 4.74 [3.88-5.70] | 4.71 [3.89-5.61] | 4.78 [3.84-5.76] | 0.07 | 0.772 |
| IL-10RB | 6.09 [5.89-6.25] | 6.07 [5.88-6.23] | 6.09 [5.90-6.26] | 0.02 | 0.773 |
| ADA | 4.54 [4.30-4.79] | 4.54 [4.30-4.77] | 4.54 [4.29-4.81] | 0.00 | 0.801 |
| IL-6 | 3.38 [2.91-3.80] | 3.42 [2.88-3.74] | 3.36 [2.94-3.84] | -0.06 | 0.801 |
| FGF-19 | 7.91 [7.10-8.57] | 7.95 [6.80-8.62] | 7.88 [7.19-8.41] | -0.07 | 0.858 |
| CX3CL1 | 6.51 [6.23-6.77] | 6.52 [6.23-6.75] | 6.49 [6.24-6.77] | -0.03 | 0.867 |
| CCL19 | 9.69 [9.29-10.1] | 9.68 [9.30-10.2] | 9.73 [9.28-10.1] | 0.05 | 0.914 |
| MCP-4 | 13.7 [13.3-14.2] | 13.7 [13.3-14.2] | 13.7 [13.2-14.1] | 0.02 | 0.948 |
| ARTN | 1.52 [1.21-1.92] | 1.54 [1.17-2.12] | 1.52 [1.29-1.77] | -0.02 | 0.954 |
| IL-17A | 1.96 [1.66-2.32] | 1.96 [1.65-2.31] | 1.96 [1.67-2.32] | 0.00 | 0.957 |
| ST1A1 | 4.12 [3.56-4.55] | 4.14 [3.57-4.54] | 4.11 [3.56-4.56] | -0.03 | 0.976 |

Data are presented as medians with interquartile ranges [IQR]. *P*-values ≤0.05 were considered to be nominally statistically significant. ^†^Difference in protein concentration (NPX) between patients with above- and below-median fatigue scores.^*^Nominal *P*-values, which all did not reach statistical significance after adjusting for multiple comparisons (using the Benjamini-Hochberg procedure, all proteins having a false discovery rate [FDR]>0.1).

**Supplementary Table S4**. Differences in plasma protein concentrations (normalized protein expression, NPX) between mildly fatigued patients (Q1 of fatigue scores) and severely fatigued patients (Q4 of fatigue scores) among patients with CD.

| **Protein** | **Total** | **Q1 fatigue score (0-3)** | **Q4 fatigue score (7-10)** | **Δ Q4-Q1** | ***P*-value** |
| --- | --- | --- | --- | --- | --- |
|  | *n*=88 | *n*=48 | *n*=40 |  |  |
| EN-RAGE | 2.45 [2.05-2.87] | 2.61 [2.24-2.96] | 2.27 [1.88-2.61] | -0.34 | 0.009 |
| GDNF | 2.66 [2.49-2.98] | 2.76 [2.56-3.09] | 2.63 [2.37-2.94] | -0.13 | 0.046 |
| IL-20RA | 1.67 [1.51-1.84] | 1.63 [1.50-1.72] | 1.76 [1.52-2.09] | 0.13 | 0.051 |
| CD40 | 12.0 [11.7-12.4] | 11.8 [11.7-12.2] | 12.1 [11.8-12.5] | 0.32 | 0.052 |
| CXCL1 | 10.9 [10.5-11.4] | 10.8 [10.4-11.2] | 11.1 [10.6-11.5] | 0.33 | 0.058 |
| CXCL10 | 9.71 [9.09-10.4] | 9.96 [9.40-10.6] | 9.44 [9.03-10.1] | -0.52 | 0.076 |
| CXCL9 | 7.16 [6.49-7.69] | 7.29 [6.70-7.82] | 6.72 [6.37-7.50] | -0.57 | 0.088 |
| SIRT2 | 7.21 [6.62-8.13] | 6.98 [6.53-7.76] | 7.57 [6.75-8.46] | 0.59 | 0.104 |
| β-NGF | 1.46 [1.36-1.65] | 1.51 [1.39-1.67] | 1.42 [1.36-1.60] | -0.09 | 0.117 |
| IL-18 | 8.54 [8.16-8.96] | 8.66 [8.30-9.01] | 8.42 [7.99-8.91] | -0.24 | 0.119 |
| SLAMF1 | 3.13 [2.87-3.44] | 3.24 [2.82-3.49] | 3.02 [2.90-3.17] | -0.22 | 0.123 |
| CD5 | 4.64 [4.40-4.80] | 4.60 [4.38-4.79] | 4.71 [4.46-4.82] | 0.11 | 0.131 |
| IL-15RA | 0.96 [0.85-1.09] | 1.00 [0.88-1.12] | 0.95 [0.77-1.08] | -0.05 | 0.131 |
| STAMBP | 7.33 [6.75-8.09] | 7.10 [6.72-7.88] | 7.83 [6.79-8.56] | 0.73 | 0.136 |
| NT-3 | 3.19 [2.98-3.65] | 3.10 [2.93-3.52] | 3.27 [3.06-3.82] | 0.17 | 0.140 |
| LIF-R | 3.59 [3.42-3.74] | 3.60 [3.49-3.75] | 3.50 [3.37-3.71] | -0.10 | 0.147 |
| TNFSF14 | 4.28 [3.96-4.72] | 4.24 [3.81-4.53] | 4.42 [4.09-4.82] | 0.18 | 0.149 |
| SCF | 9.68 [9.49-9.95] | 9.73 [9.54-9.98] | 9.64 [9.25-9.90] | -0.09 | 0.159 |
| ADA | 4.57 [4.31-4.84] | 4.52 [4.27-4.78] | 4.62 [4.35-5.04] | 0.10 | 0.162 |
| IL-7 | 3.31 [2.90-3.61] | 3.23 [2.76-3.57] | 3.44 [2.95-3.87] | 0.21 | 0.172 |
| AXIN1 | 6.20 [5.51-7.15] | 6.07 [5.45-6.95] | 6.50 [5.58-7.25] | 0.43 | 0.177 |
| DNER | 9.21 [9.01-9.38] | 9.27 [9.01-9.39] | 9.13 [9.01-9.34] | -0.14 | 0.180 |
| TRANCE | 4.95 [4.48-5.34] | 5.02 [4.55-5.41] | 4.84 [4.45-5.21] | -0.18 | 0.200 |
| ARTN | 1.36 [1.15-1.81] | 1.28 [1.14-1.57] | 1.60 [1.28-1.97] | 0.32 | 0.205 |
| IFN-γ | 6.49 [5.51-7.46] | 6.61 [5.54-7.58] | 6.22 [5.49-7.10] | -0.39 | 0.206 |
| CCL4 | 5.98 [5.54-6.35] | 6.09 [5.65-6.35] | 5.77 [5.50-6.35] | -0.32 | 0.224 |
| MCP-1 | 10.9 [10.7-11.2] | 10.9 [10.7-11.3] | 10.9 [10.5-11.1] | -0.02 | 0.224 |
| VEGF-A | 10.0 [9.70-10.3] | 9.91 [9.67-10.3] | 10.1 [9.85-10.4] | 0.19 | 0.239 |
| CXCL5 | 11.6 [11.0-12.4] | 11.5 [11.0-12.2] | 11.8 [11.0-12.7] | 0.25 | 0.272 |
| CCL3 | 5.45 [5.09-5.79] | 5.49 [5.13-5.81] | 5.39 [4.95-5.66] | -0.10 | 0.283 |
| IL-10RA | 1.16 [1.00-1.72] | 1.10 [0.99-1.69] | 1.38 [1.02-2.06] | 0.28 | 0.292 |
| IL-10RB | 6.06 [5.88-6.25] | 6.09 [5.89-6.29] | 6.03 [5.88-6.19] | -0.06 | 0.307 |
| CASP-8 | 3.21 [2.70-3.85] | 3.35 [2.65-4.02] | 3.12 [2.74-3.55] | -0.23 | 0.335 |
| IL-4 | 1.70 [1.29-2.43] | 1.81 [1.41-3.62] | 1.55 [1.27-2.17] | -0.26 | 0.336 |
| CD6 | 5.18 [4.92-5.50] | 5.17 [4.92-5.42] | 5.33 [4.93-5.59] | 0.16 | 0.348 |
| IL-10 | 4.01 [3.67-4.38] | 4.06 [3.63-4.52] | 3.96 [3.67-4.17] | -0.10 | 0.352 |
| CCL28 | 2.43 [2.22-2.72] | 2.42 [2.21-2.69] | 2.46 [2.24-2.74] | 0.04 | 0.361 |
| MCP-2 | 9.34 [8.94-9.66] | 9.24 [8.94-9.58] | 9.40 [9.00-9.76] | 0.16 | 0.365 |
| uPA | 9.74 [9.48-9.96] | 9.70 [9.41-9.97] | 9.79 [9.58-9.96] | 0.09 | 0.374 |
| LAP TGF-β-1 | 7.59 [7.27-7.93] | 7.54 [7.21-7.81] | 7.67 [7.31-8.02] | 0.13 | 0.383 |
| 4E-BP1 | 8.96 [8.42-9.51] | 8.88 [8.38-9.49] | 9.05 [8.55-9.63] | 0.17 | 0.390 |
| IL-13 | 2.34 [1.73-3.61] | 2.62 [1.89-3.70] | 1.79 [1.73-3.00] | -0.83 | 0.402 |
| CST5 | 5.89 [5.59-6.21] | 5.93 [5.63-6.17] | 5.81 [5.52-6.25] | -0.12 | 0.412 |
| OPG | 10.2 [9.89-10.5] | 10.1 [9.9-10.4] | 10.2 [9.91-10.5] | 0.11 | 0.416 |
| MCP-3 | 1.90 [1.69-2.10] | 1.89 [1.59-2.09] | 1.93 [1.73-2.13] | 0.04 | 0.425 |
| FGF-21 | 5.10 [4.15-5.86] | 5.15 [4.38-5.87] | 5.08 [4.09-5.73] | -0.07 | 0.495 |
| TRAIL | 8.67 [8.46-8.90] | 8.74 [8.50-8.90] | 8.64 [8.44-8.90] | -0.10 | 0.497 |
| IL-17A | 1.93 [1.62-2.29] | 1.95 [1.63-2.31] | 1.93 [1.53-2.28] | -0.02 | 0.498 |
| IL-2RB | 2.01 [1.76-2.61] | 2.01 [1.70-2.48] | 2.16 [1.75-3.15] | 0.15 | 0.501 |
| TGF-α | 4.14 [3.97-4.33] | 4.21 [3.97-4.34] | 4.06 [3.97-4.32] | -0.15 | 0.552 |
| IL-5 | 2.43 [1.86-4.08] | 2.43 [1.86-4.59] | 2.44 [1.88-3.27] | 0.01 | 0.555 |
| ST1A1 | 4.14 [3.50-4.53] | 4.12 [3.47-4.48] | 4.18 [3.65-4.55] | 0.06 | 0.556 |
| CCL11 | 7.17 [6.84-7.55] | 7.19 [6.84-7.55] | 7.09 [6.84-7.57] | -0.10 | 0.557 |
| IL-18R1 | 8.43 [8.11-8.63] | 8.41 [8.19-8.65] | 8.47 [7.98-8.63] | 0.06 | 0.563 |
| CD244 | 7.10 [6.89-7.51] | 7.10 [6.88-7.41] | 7.13 [6.91-7.81] | 0.03 | 0.569 |
| IL-8 | 5.66 [5.30-6.07] | 5.68 [5.32-6.10] | 5.65 [5.21-6.02] | -0.03 | 0.598 |
| MMP-10 | 6.59 [6.30-6.87] | 6.60 [6.40-6.91] | 6.58 [6.21-6.83] | -0.02 | 0.598 |
| TNFRSF9 | 6.63 [6.37-6.96] | 6.63 [6.41-6.99] | 6.62 [6.28-6.96] | -0.01 | 0.615 |
| CXCL11 | 8.46 [7.93-9.23] | 8.52 [7.99-9.03] | 8.34 [7.86-9.37] | -0.18 | 0.621 |
| IL-6 | 3.42 [2.81-3.89] | 3.49 [2.92-3.79] | 3.22 [2.77-4.08] | -0.27 | 0.645 |
| IL-12B | 6.39 [5.97-6.84] | 6.40 [5.95-6.78] | 6.33 [5.98-6.95] | -0.07 | 0.681 |
| MMP-1 | 10.1 [9.52-10.9] | 10.1 [9.58-11.1] | 10.2 [9.39-10.8] | 0.08 | 0.694 |
| CCL19 | 9.75 [9.37-10.1] | 9.72 [9.40-10.1] | 9.80 [9.36-10.2] | 0.08 | 0.712 |
| PD-L1 | 7.03 [6.72-7.25] | 7.04 [6.82-7.25] | 7.01 [6.68-7.31] | -0.03 | 0.750 |
| TNFB | 4.85 [4.54-5.06] | 4.88 [4.59-5.06] | 4.80 [4.53-5.06] | -0.08 | 0.750 |
| FGF-19 | 7.75 [6.63-8.45] | 7.54 [6.58-8.52] | 7.91 [6.99-8.40] | 0.37 | 0.769 |
| CCL25 | 5.82 [5.41-6.27] | 5.75 [5.44-6.21] | 5.92 [5.22-6.37] | 0.17 | 0.789 |
| CD8A | 10.0 [9.7-10.6] | 10.0 [9.71-10.6] | 10.1 [9.42-10.6] | 0.05 | 0.795 |
| HGF | 8.82 [8.58-9.23] | 8.82 [8.60-9.28] | 8.86 [8.49-9.19] | 0.04 | 0.795 |
| CCL23 | 9.95 [9.65-10.3] | 9.95 [9.67-10.2] | 9.99 [9.63-10.4] | 0.04 | 0.802 |
| CCL20 | 6.82 [6.51-7.46] | 6.83 [6.51-7.69] | 6.78 [6.51-7.31] | -0.05 | 0.828 |
| CXCL6 | 8.82 [8.42-9.41] | 8.81 [8.44-9.41] | 8.95 [8.36-9.41] | 0.14 | 0.857 |
| IL-17C | 1.93 [1.72-2.06] | 1.87 [1.74-2.08] | 1.99 [1.69-2.05] | 0.12 | 0.873 |
| TWEAK | 9.73 [9.51-9.97] | 9.68 [9.53-10.0] | 9.77 [9.45-9.97] | 0.09 | 0.873 |
| FGF-23 | 3.41 [3.17-3.80] | 3.39 [3.18-3.79] | 3.43 [3.16-3.80] | 0.04 | 0.880 |
| CX3CL1 | 6.51 [6.25-6.86] | 6.52 [6.30-6.81] | 6.49 [6.24-6.94] | -0.03 | 0.890 |
| MCP-4 | 13.6 [13.3-14.1] | 13.6 [13.3-14.2] | 13.8 [13.2-14.1] | 0.15 | 0.893 |
| NRTN | 2.35 [1.62-2.99] | 2.37 [1.51-3.59] | 2.33 [1.66-2.93] | -0.04 | 0.947 |
| Flt3L | 9.38 [8.93-9.79] | 9.38 [8.95-9.73] | 9.37 [8.90-9.87] | -0.01 | 0.953 |
| CDCP1 | 3.28 [2.91-3.92] | 3.25 [2.88-3.92] | 3.33 [2.91-3.91] | 0.08 | 0.960 |
| OSM | 4.61 [3.94-5.40] | 4.59 [3.84-5.62] | 4.65 [4.05-5.32] | 0.06 | 0.960 |
| CSF-1 | 10.3 [10.2-10.5] | 10.3 [10.2-10.5] | 10.3 [10.2-10.5] | 0.04 | 0.987 |
| IL-24 | 1.83 [1.79-2.27] | 1.83 [1.77-2.84] | 1.88 [1.80-NA] | 0.05 | 1.000 |

**Supplementary Table S5**. Differences in plasma protein concentrations (normalized protein expression, NPX) between mildly fatigued patients (Q1 of fatigue scores) and severely fatigued patients (Q4 of fatigue scores) among patients with UC.

| **Protein** | **Total** | **Q1 fatigue score (0-3)** | **Q4 fatigue score (6-10)** | **Δ Q4-Q1** | ***P*-value** |
| --- | --- | --- | --- | --- | --- |
|  | *n*=84 | *n*=48 | *n*=36 |  |  |
| NRTN | 1.53 [1.41-2.07] | 1.49 [1.34-1.52] | 1.91 [1.53-2.94] | 0.42 | 0.025 |
| ADA | 4.52 [4.25-4.66] | 4.60 [4.30-4.83] | 4.40 [4.22-4.61] | -0.20 | 0.047 |
| FGF-23 | 3.33 [3.17-3.60] | 3.25 [3.14-3.55] | 3.41 [3.21-3.66] | 0.16 | 0.048 |
| CASP-8 | 3.29 [2.82-3.63] | 3.40 [2.87-3.71] | 3.21 [2.77-3.41] | -0.19 | 0.051 |
| IL-10RB | 6.06 [5.86-6.26] | 6.04 [5.82-6.19] | 6.17 [5.98-6.32] | 0.13 | 0.061 |
| NT-3 | 3.22 [2.96-3.53] | 3.26 [3.10-3.53] | 3.08 [2.84-3.51] | -0.16 | 0.061 |
| LIF-R | 3.55 [3.41-3.68] | 3.60 [3.44-3.71] | 3.46 [3.35-3.66] | -0.14 | 0.068 |
| CD8A | 10.0 [9.62-10.6] | 10.2 [9.67-10.7] | 9.92 [9.53-10.4] | -0.30 | 0.081 |
| CD5 | 4.68 [4.40-4.95] | 4.64 [4.34-4.87] | 4.77 [4.54-5.08] | 0.13 | 0.088 |
| CCL3 | 5.46 [5.16-5.91] | 5.39 [5.16-5.86] | 5.74 [5.15-6.04] | 0.35 | 0.116 |
| IL-13 | 2.39 [1.87-2.84] | 2.09 [1.77-3.50] | 3.26 [2.47-4.27] | 1.17 | 0.161 |
| IL-10RA | 1.21 [0.97-2.06] | 1.14 [0.94-1.94] | 1.45 [1.05-2.29] | 0.31 | 0.164 |
| CCL28 | 2.37 [2.26-2.64] | 2.45 [2.22-2.67] | 2.34 [2.26-2.55] | -0.11 | 0.175 |
| FGF-21 | 4.37 [3.36-5.02] | 4.34 [3.32-4.81] | 4.41 [3.51-5.59] | 0.07 | 0.175 |
| uPA | 9.71 [9.58-9.91] | 9.73 [9.65-9.92] | 9.71 [9.39-9.89] | -0.02 | 0.178 |
| MCP-3 | 1.95 [1.74-2.18] | 1.89 [1.65-2.11] | 1.97 [1.86-2.19] | 0.08 | 0.219 |
| IL-17A | 1.94 [1.68-2.24] | 1.86 [1.56-2.23] | 1.98 [1.79-2.24] | 0.12 | 0.237 |
| DNER | 9.14 [8.97-9.38] | 9.20 [8.97-9.40] | 9.10 [8.97-9.36] | -0.10 | 0.259 |
| IL-12B | 6.37 [5.87-6.91] | 6.24 [5.84-6.76] | 6.53 [5.91-6.96] | 0.29 | 0.266 |
| OPG | 10.0 [9.81-10.3] | 10.0 [9.81-10.2] | 10.0 [9.81-10.5] | -0.01 | 0.278 |
| IL-15RA | 1.00 [0.86-1.18] | 0.97 [0.86-1.14] | 1.02 [0.91-1.22] | 0.05 | 0.280 |
| IL-2RB | 1.81 [1.70-2.04] | 1.83 [1.72-2.16] | 1.73 [1.67-1.88] | -0.10 | 0.301 |
| TWEAK | 9.69 [9.49-9.90] | 9.76 [9.53-9.91] | 9.64 [9.33-9.88] | -0.12 | 0.307 |
| CCL11 | 7.36 [6.99-7.73] | 7.42 [7.07-7.75] | 7.24 [6.84-7.73] | -0.18 | 0.329 |
| IL-18 | 8.54 [8.21-8.88] | 8.66 [8.27-8.92] | 8.48 [8.18-8.86] | -0.18 | 0.333 |
| GDNF | 2.74 [2.46-2.95] | 2.74 [2.49-2.95] | 2.74 [2.21-2.96] | 0.00 | 0.355 |
| TRAIL | 8.71 [8.51-8.92] | 8.66 [8.47-8.93] | 8.80 [8.58-8.92] | 0.14 | 0.376 |
| VEGF-A | 9.93 [9.66-10.1] | 9.90 [9.66-10.1] | 9.99 [9.67-10.2] | 0.09 | 0.391 |
| CSF-1 | 10.3 [10.1-10.4] | 10.3 [10.1-10.4] | 10.3 [10.2-10.4] | 0.04 | 0.396 |
| TGF-α | 4.14 [3.98-4.33] | 4.12 [3.91-4.34] | 4.17 [4.06-4.33] | 0.05 | 0.401 |
| STAMBP | 7.29 [6.49-8.03] | 7.39 [6.59-8.13] | 7.13 [6.40-7.77] | -0.26 | 0.406 |
| IL-17C | 1.85 [1.71-2.27] | 1.85 [1.74-2.25] | 1.85 [1.69-2.27] | 0.00 | 0.434 |
| CCL4 | 5.96 [5.56-6.34] | 5.96 [5.50-6.25] | 5.86 [5.56-6.41] | -0.10 | 0.445 |
| ST1A1 | 4.08 [3.50-4.60] | 4.14 [3.58-4.67] | 4.07 [3.39-4.46] | -0.07 | 0.453 |
| SIRT2 | 7.11 [6.42-7.98] | 7.21 [6.44-8.09] | 7.02 [6.25-7.92] | -0.19 | 0.472 |
| CX3CL1 | 6.56 [6.28-6.79] | 6.49 [6.23-6.79] | 6.58 [6.34-6.86] | 0.09 | 0.492 |
| IL-7 | 3.22 [2.87-3.61] | 3.29 [2.90-3.61] | 3.20 [2.70-3.62] | -0.09 | 0.492 |
| 4E-BP1 | 8.95 [8.14-9.51] | 8.98 [8.18-9.64] | 8.74 [8.08-9.48] | -0.24 | 0.515 |
| HGF | 8.77 [8.58-9.03] | 8.77 [8.62-9.03] | 8.77 [8.49-9.03] | 0.00 | 0.527 |
| AXIN1 | 6.08 [5.21-6.96] | 6.16 [5.21-6.98] | 6.00 [5.00-6.76] | -0.16 | 0.539 |
| CXCL6 | 8.71 [8.04-9.35] | 8.89 [8.12-9.35] | 8.63 [8.01-9.39] | -0.26 | 0.551 |
| CXCL11 | 8.46 [7.89-9.24] | 8.44 [7.90-9.10] | 8.72 [7.80-9.52] | 0.28 | 0.557 |
| FGF-19 | 8.31 [7.52-8.85] | 8.31 [7.52-8.65] | 8.27 [7.47-9.13] | -0.04 | 0.569 |
| CST5 | 5.93 [5.64-6.28] | 5.90 [5.57-6.28] | 5.95 [5.72-6.30] | 0.05 | 0.575 |
| CCL19 | 9.61 [9.17-10.1] | 9.61 [9.24-10.2] | 9.61 [9.01-10.1] | 0.00 | 0.594 |
| IL-18R1 | 8.24 [7.94-8.62] | 8.24 [7.86-8.62] | 8.24 [8.03-8.60] | 0.00 | 0.613 |
| CCL20 | 6.75 [6.29-7.35] | 6.68 [6.44-7.32] | 6.89 [6.24-7.39] | 0.21 | 0.625 |
| CXCL9 | 7.47 [6.93-8.01] | 7.47 [6.93-7.97] | 7.46 [6.94-8.62] | -0.01 | 0.625 |
| IL-10 | 4.29 [3.74-4.93] | 4.32 [3.68-4.93] | 4.26 [3.78-5.35] | -0.06 | 0.625 |
| SCF | 9.69 [9.38-9.83] | 9.69 [9.32-9.86] | 9.69 [9.44-9.81] | 0.00 | 0.632 |
| MMP-10 | 6.68 [6.26-7.08] | 6.63 [6.22-7.10] | 6.74 [6.26-7.07] | 0.11 | 0.651 |
| IL-24 | 2.17 [1.88-2.27] | 2.18 [1.88-2.52] | 2.01 [1.84-NA] | -0.17 | 0.665 |
| IL-20RA | 1.62 [1.51-1.77] | 1.62 [1.52-1.72] | 1.64 [1.49-1.83] | 0.02 | 0.700 |
| TNFSF14 | 4.22 [3.81-4.63] | 4.21 [3.78-4.63] | 4.23 [3.93-4.66] | 0.02 | 0.728 |
| IFN-γ | 6.19 [5.44-7.02] | 6.11 [5.46-6.91] | 6.40 [5.44-7.15] | 0.29 | 0.738 |
| MMP-1 | 10.1 [9.21-10.9] | 10.2 [9.18-10.9] | 9.98 [9.22-10.9] | -0.23 | 0.745 |
| CDCP1 | 3.51 [3.20-4.01] | 3.49 [3.21-3.90] | 3.52 [3.20-4.14] | 0.03 | 0.752 |
| IL-8 | 5.58 [5.21-5.94] | 5.57 [5.32-5.94] | 5.63 [5.18-5.99] | 0.06 | 0.800 |
| SLAMF1 | 2.98 [2.70-3.37] | 2.96 [2.71-3.33] | 3.02 [2.67-3.38] | 0.06 | 0.807 |
| CCL25 | 6.07 [5.63-6.46] | 6.02 [5.65-6.49] | 6.13 [5.44-6.46] | 0.11 | 0.814 |
| CXCL5 | 11.4 [10.5-12.3] | 11.4 [10.6-12.3] | 11.4 [10.4-12.3] | 0.04 | 0.814 |
| MCP-4 | 13.6 [13.2-14.2] | 13.5 [13.2-14.2] | 13.6 [13.1-14.2] | 0.10 | 0.821 |
| TNFRSF9 | 6.73 [6.39-7.01] | 6.74 [6.43-6.92] | 6.71 [6.34-7.08] | -0.03 | 0.821 |
| CXCL10 | 9.95 [9.45-10.5] | 9.94 [9.61-10.6] | 9.95 [9.36-10.5] | 0.01 | 0.828 |
| TRANCE | 4.85 [4.40-5.32] | 4.84 [4.33-5.45] | 4.87 [4.54-5.22] | 0.03 | 0.828 |
| CD244 | 7.14 [6.89-7.53] | 7.09 [6.89-7.55] | 7.32 [6.85-7.50] | 0.23 | 0.835 |
| β-NGF | 1.49 [1.37-1.61] | 1.50 [1.32-1.65] | 1.46 [1.39-1.60] | -0.04 | 0.842 |
| CXCL1 | 10.7 [10.1-11.2] | 10.7 [10.2-11.2] | 10.7 [10.1-11.2] | 0.07 | 0.842 |
| LAP TGF-β-1 | 7.53 [7.19-7.82] | 7.54 [7.25-7.80] | 7.53 [7.06-7.86] | -0.01 | 0.842 |
| OSM | 4.60 [3.96-5.28] | 4.58 [3.85-5.26] | 4.61 [3.99-5.29] | 0.03 | 0.849 |
| IL-6 | 3.17 [2.81-3.59] | 3.18 [2.78-3.56] | 3.16 [2.85-3.70] | -0.02 | 0.871 |
| CCL23 | 9.91 [9.65-10.2] | 9.91 [9.65-10.2] | 9.88 [9.67-10.2] | -0.03 | 0.878 |
| PD-L1 | 7.03 [6.70-7.25] | 7.03 [6.62-7.34] | 7.03 [6.79-7.21] | 0.00 | 0.878 |
| ARTN | 1.61 [1.18-2.41] | 1.66 [1.17-2.42] | 1.40 [1.25-2.29] | -0.26 | 0.882 |
| IL-5 | 2.29 [1.88-2.65] | 2.30 [1.90-2.93] | 2.28 [1.84-2.56] | -0.02 | 0.885 |
| EN-RAGE | 2.51 [2.20-2.91] | 2.60 [2.18-3.15] | 2.49 [2.25-2.89] | -0.11 | 0.892 |
| CD6 | 5.29 [4.98-5.61] | 5.26 [4.94-5.56] | 5.29 [4.99-5.69] | 0.03 | 0.899 |
| TNFB | 4.87 [4.51-5.15] | 4.85 [4.51-5.15] | 4.90 [4.49-5.17] | 0.05 | 0.906 |
| IL-4 | 1.72 [1.38-2.74] | 1.72 [1.38-2.74] | 1.96 [1.35-3.29] | 0.24 | 0.932 |
| Flt3L | 9.32 [8.93-9.60] | 9.32 [9.02-9.58] | 9.32 [8.91-9.65] | 0.00 | 0.950 |
| MCP-2 | 9.25 [8.80-9.57] | 9.26 [8.62-9.57] | 9.24 [8.81-9.61] | -0.02 | 0.950 |
| CD40 | 12.0 [11.5-12.3] | 11.9 [11.6-12.2] | 12.0 [11.4-12.3] | 0.05 | 0.957 |
| MCP-1 | 10.8 [10.6-11.1] | 10.9 [10.6-11.1] | 10.8 [10.5-11.3] | -0.07 | 0.986 |

**Supplementary Table S6**. Abbreviations, full names, UniProt IDs and detection rates of all 92 plasma proteins measured using the Olink^Ⓡ^ Inflammation panel.

| **Abbreviation** | **Full name** | **UniProt ID** | **% IBD** | **% CD** | **% UC** |
| --- | --- | --- | --- | --- | --- |
|  |  |  | *n*=350 | *n*=188 | *n*=162 |
| 4E-BP1 | Eukaryotic translation initiation factor 4E-binding protein 1 | Q13541 | 100 | 100 | 100 |
| ADA | Adenosine Deaminase | P00813 | 100 | 100 | 100 |
| ARTN | Artemin | Q5T4W7 | 21.7 | 22.9 | 20.4 |
| AXIN1 | Axin-1 | O15169 | 100 | 100 | 100 |
| β-NGF | Beta-nerve growth factor | P01138 | 100 | 100 | 100 |
| CASP-8 | Caspase-8 | Q14790 | 100 | 100 | 100 |
| CCL11 | Eotaxin | P51671 | 100 | 100 | 100 |
| CCL19 | C-C motif chemokine 19 | Q99731 | 100 | 100 | 100 |
| CCL20 | C-C motif chemokine 20 | P78556 | 100 | 100 | 100 |
| CCL23 | C-C motif chemokine 23 | P55773 | 100 | 100 | 100 |
| CCL25 | C-C motif chemokine 25 | O15444 | 100 | 100 | 100 |
| CCL28 | C-C motif chemokine 28 | Q9NRJ3 | 100 | 100 | 100 |
| CCL3 | C-C motif chemokine 3 | P10147 | 100 | 100 | 100 |
| CCL4 | C-C motif chemokine 4 | P13236 | 100 | 100 | 100 |
| CD244 | Natural killer cell receptor 2B4 | Q9BZW8 | 100 | 100 | 100 |
| CD40 | CD40L receptor | P25942 | 100 | 100 | 100 |
| CD5 | T-cell surface glycoprotein CD5 | P06127 | 100 | 100 | 100 |
| CD6 | T-cell surface glycoprotein CD6 isoform | P30203 | 100 | 100 | 100 |
| CD8A | T-cell surface glycoprotein CD8 alpha chain | P01732 | 100 | 100 | 100 |
| CDCP1 | CUB domain-containing protein 1 | Q9H5V8 | 100 | 100 | 100 |
| CSF-1 | Macrophage colony-stimulating factor 1 | P09603 | 100 | 100 | 100 |
| CST5 | Cystatin D | P28325 | 100 | 100 | 100 |
| CX3CL1 | Fractalkine | P78423 | 100 | 100 | 100 |
| CXCL1 | C-X-C motif chemokine 1 | P09341 | 100 | 100 | 100 |
| CXCL10 | C-X-C motif chemokine 10 | P02778 | 100 | 100 | 100 |
| CXCL11 | C-X-C motif chemokine 11 | O14625 | 100 | 100 | 100 |
| CXCL5 | C-X-C motif chemokine 5 | P42830 | 100 | 100 | 100 |
| CXCL6 | C-X-C motif chemokine 6 | P80162 | 100 | 100 | 100 |
| CXCL9 | C-X-C motif chemokine 9 | Q07325 | 100 | 100 | 100 |
| DNER | Delta and Notch-like epidermal growth factor-related receptor | Q8NFT8 | 100 | 100 | 100 |
| EN-RAGE | Protein S100-A12 | P80511 | 99.4 | 98.9 | 100 |
| FGF-5 | Fibroblast growth factor 5 | P12034 | 0 | 0 | 0 |
| FGF-19 | Fibroblast growth factor 19 | O95750 | 100 | 100 | 100 |
| FGF-21 | Fibroblast growth factor 21 | Q9NSA1 | 98.9 | 99.5 | 98.1 |
| FGF-23 | Fibroblast growth factor 23 | Q9GZV9 | 98.9 | 100 | 97.5 |
| Flt3L | Fms-related tyrosine kinase 3 ligand | P49771 | 100 | 100 | 100 |
| GDNF | Glial cell line-derived neurotrophic factor | P39905 | 98.3 | 98.4 | 98.1 |
| HGF | Hepatocyte growth factor | P14210 | 100 | 100 | 100 |
| IFN-γ | Interferon gamma | P01579 | 99.4 | 100 | 98.8 |
| IL-1α | Interleukin-1 alpha | P01583 | 0 | 0 | 0 |
| IL-10 | Interleukin-10 | P22301 | 100 | 100 | 100 |
| IL-10RA | Interleukin-10 receptor subunit alpha | Q13651 | 63.1 | 63.3 | 63.0 |
| IL-10RB | Interleukin-10 receptor subunit beta | Q08334 | 100 | 100 | 100 |
| IL-12B | Interleukin-12 subunit beta | P29460 | 100 | 100 | 100 |
| IL-13 | Interleukin-13 | P35225 | 21.7 | 18.1 | 25.9 |
| IL-15RA | Interleukin-15 receptor subunit alpha | Q13261 | 94.9 | 93.6 | 96.3 |
| IL-17A | Interleukin-17A | Q16552 | 92.3 | 93.6 | 90.7 |
| IL-17C | Interleukin-17C | Q9P0M4 | 49.1 | 48.9 | 49.4 |
| IL-18 | Interleukin-18 | Q14116 | 100 | 100 | 100 |
| IL-18R1 | Interleukin-18 receptor 1 | Q13478 | 100 | 100 | 100 |
| IL-2 | Interleukin-2 | P60568 | 0 | 0 | 0 |
| IL-20 | Interleukin-20 | Q9NYY1 | 0 | 0 | 0 |
| IL-20RA | Interleukin-20 receptor subunit alpha | Q9UHF4 | 48.0 | 52.1 | 43.2 |
| IL-22RA1 | Interleukin-22 receptor subunit alpha-1 | Q8N6P7 | 0 | 0 | 0 |
| IL-24 | Interleukin-24 | Q13007 | 13.4 | 13.3 | 13.6 |
| IL-2RB | Interleukin-2 receptor subunit beta | P14784 | 21.7 | 20.2 | 23.5 |
| IL-33 | Interleukin-33 | O95760 | 0 | 0 | 0 |
| IL-4 | Interleukin-4 | P05112 | 18.9 | 14.9 | 23.5 |
| IL-5 | Interleukin-5 | P05113 | 42.3 | 45.2 | 38.9 |
| IL-6 | Interleukin-6 | P05231 | 100 | 100 | 100 |
| IL-7 | Interleukin-7 | P13232 | 100 | 100 | 100 |
| IL-8 | Interleukin-8 | P10145 | 100 | 100 | 100 |
| LAP TGF-β-1 | Latency-associated peptide transforming growth factor beta-1 | P01137 | 100 | 100 | 100 |
| LIF | Leukemia inhibitory factor | P15018 | 0 | 0 | 0 |
| LIF-R | Leukemia inhibitory factor receptor | P42702 | 99.7 | 99.5 | 100 |
| MCP-1 | Monocyte chemotactic protein 1 | P13500 | 100 | 100 | 100 |
| MCP-2 | Monocyte chemotactic protein 2 | P80075 | 100 | 100 | 100 |
| MCP-3 | Monocyte chemotactic protein 3 | P80098 | 70.0 | 71.8 | 67.9 |
| MCP-4 | Monocyte chemotactic protein 4 | Q99616 | 100 | 100 | 100 |
| MMP-1 | Matrix metalloproteinase-1 | P03956 | 100 | 100 | 100 |
| MMP-10 | Matrix metalloproteinase-10 | P09238 | 100 | 100 | 100 |
| NRTN | Neurturin | Q99748 | 12.9 | 13.3 | 12.3 |
| NT-3 | Neurotrophin-3 | P20783 | 100 | 100 | 100 |
| OPG | Osteoprotegerin | O00300 | 100 | 100 | 100 |
| OSM | Oncostatin-M | P13725 | 100 | 100 | 100 |
| PD-L1 | Programmed cell death 1 ligand 1 | Q9NZQ7 | 100 | 100 | 100 |
| SCF | Stem cell factor | P21583 | 100 | 100 | 100 |
| SIRT2 | SIR2-like protein 2 | Q8IXJ6 | 100 | 100 | 100 |
| SLAMF1 | Signaling lymphocytic activation molecule | Q13291 | 100 | 100 | 100 |
| ST1A1 | Sulfotransferase 1A1 | P50225 | 98.9 | 98.4 | 99.4 |
| STAMBP | STAM-binding protein | O95630 | 100 | 100 | 100 |
| TGF-α | Transforming growth factor alpha | P01135 | 100 | 100 | 100 |
| TNF | Tumor necrosis factor | P01375 | 0 | 0 | 0 |
| TNFB | TNF-beta | P01374 | 100 | 100 | 100 |
| TNFRSF9 | Tumor necrosis factor receptor superfamily member 9 | Q07011 | 100 | 100 | 100 |
| TNFSF14 | Tumor necrosis factor ligand superfamily member 14 | O43557 | 100 | 100 | 100 |
| TRAIL | TNF-related apoptosis-inducing ligand | P50591 | 100 | 100 | 100 |
| TRANCE | TNF-related activation-induced cytokine | O14788 | 100 | 100 | 100 |
| TSLP | Thymic stromal lymphopoietin | Q969D9 | 0 | 0 | 0 |
| TWEAK | Tumor necrosis factor (Ligand) superfamily, member 12 | O43508 | 100 | 100 | 100 |
| uPA | Urokinase-type plasminogen activator | P00749 | 100 | 100 | 100 |
| VEGF-A | Vascular endothelial growth factor A | P15692 | 100 | 100 | 100 |
